# A Guide for Businesses and Employers Responding to Novel Coronavirus disease (COVID‐19): 4th edition

**DOI:** 10.1002/1348-9585.12225

**Published:** 2021-10-29

**Authors:** Hidetaka Suzuki, Toshiaki Miyamoto, Atsuo Hamada, Akiyasu Nakano, Hirofumi Okoshi, Fumihiro Yamasawa, Members of Occupational Health Committee on JSTH (Japanese Society of Travel and Health)

**Affiliations:** ^1^ H Consulting Services Tokyo Japan; ^2^ Japan Society for Occupational Health Tokyo Japan; ^3^ Tokyo Medical University Hospital Tokyo Japan; ^4^ Marunouchi Sogo Law Office Tokyo Japan; ^5^ Kojinkai Nishishinbashi Clinic Tokyo Japan; ^6^ Marubeni Corporation Tokyo Japan

**Keywords:** COVID‐19, Guide, labor management, Preventive measures, workplace

## Abstract

The Japanese Society of Travel and Health (JSTH) and the Japanese Society for Occupational Health (JSOH) have compiled “Novel Coronavirus Information” together as a joint document, which has been shared with the public on their respective websites since February 2020. In May 11, 2020, this document was to be published as “A Guide for Businesses and Employers Responding to Novel Coronavirus Disease (COVID‐19)” (hereinafter referred to as “this Guide”). This Guide was prepared for persons in charge of COVID‐19 control measures in their workplace. It should be used at the discretion of each business operator according to their workplace environment. The examples of infection control measures shown in this Guide are not guaranteed to work for all situations, and they do not limit or bind actual measures being put in place. When selecting actual measures, it is necessary to obtain new information and thoroughly understand individual cases and situations. This Guide was prepared based on findings and reports about the virus and response measures taken by the relevant ministries (Ministry of Health, Labour and Welfare, Ministry of Foreign Affairs, etc.) that could be confirmed as of December 15, 2020. Therefore, the contents of this Guide may need to be modified in the future, depending on changes in the situations mentioned above. In the preparation of this Guide, every effort has been made to ensure the accuracy of currently obtainable information. However, neither JSTH or JSOH shall be held liable for any unfavorable circumstance, such as loss and damage (including lost profits and various expenses), harmful rumors, etc. experienced by a business operator, his/her employees, and any other persons concerned as a result of various measures considered/implemented using this Guide by persons responsible for infection control in the workplace.

## 1 PREFACE TO THE FOURTH EDITION

December 15, 2020.

Japanese Society of Travel and Health President Takashi Nakano.

Japan Society for Occupational Health President Norito Kawakami.

While we seek a balance between preventing the spread of the novel coronavirus disease COVID‐19 and promoting economic recovery, a growing number of new and severe cases is being reported daily. Medical communities are assumed to go through an extremely challenging time in the end of this year. Especially in the prefectures where medical systems are facing collapse, implementation of the “Go To Travel” campaign, part of the national policy aimed at rebooting the economy, has been temporarily stopped or voluntarily suspended.

On October 23, the Subcommittee on Novel Coronavirus Disease Control within the Ministry of Health, Labour and Welfare proposed “‘5 situations’ that increase the risk of infection.” The “5 situations” are often business‐related. Many workplace clusters have also been reported, and there has been a surge of new cases resulting from household transmission. This indicates that the disease may be spreading from the workplace to the home, or from the home to the workplace. To prevent the “5 situations” from creeping into our own lives, we need to exercise caution daily and continue to take and review actions to prevent the spread of COVID‐19 in our workplaces.

Now, “cross‐border business travel based on bilateral agreements” has been resumed. With the cooperation of many medical institutions, the Ministry of Economy, Trade and Industry has launched TeCOT (COVID‐19 Testing Center for Overseas Travelers), which has been facilitating overseas travel.

Provision of the latest information through collaboration between JSTH and JSOH to help maintain/resume business operations without increasing infection risks for travelers, workplaces, and employees is considered beneficial to the Japanese people. We ask for your continued cooperation in preventing the spread of the disease.

## 2 PREFACE TO THE FIRST EDITION

May 11, 2020

Kazunobu Ouchi, President, Japanese Society of Travel and Health.

Norito Kawakami, President, Japan Society for Occupational Health.

An infectious disease caused by a novel coronavirus that emerged from Wuhan, China, in December 2019 and named COVID‐19 has rapidly spread across the globe, from China to the rest of Asia, Europe, and North America. Our country has been no exception. Starting with border control measures to prevent the influx of infected people from overseas, the central and local governments, healthcare professionals, and all citizens are now desperately working together to prevent the formation of clusters and the collapse of healthcare systems to ensure the safety and health of the Japanese people. Unfortunately, many new COVID‐19 cases continue to be reported, and the number of deaths is on the rise. We would like to extend our heartfelt sympathy to all people who have lost their lives as a result of COVID‐19. Travel medicine is a discipline encompassing general medical care associated with travel, while occupational health is a discipline aimed at supporting the safety and health of workers, workplaces, and business operators. Therefore, COVID‐19 can be considered a disease that truly requires the involvement of both Societies. Until now, JSTH Occupational Health Committee and JSOH Study Group on Health Administration for Overseas Workers have worked together in actively providing various facts and information about COVID‐19 as "Novel Coronavirus Information,” which has gone through multiple revisions according to the shifting situations in Japan and overseas. JSTH and JSOH will continue to work together compiling and updating this “Novel Coronavirus Information,” now renamed “A Guide to Novel Coronavirus Infection (COVID‐19) Control for Workplaces.” This guide may be useful not just for overseas travelers but as a source of measures in general occupational environments, such as for workplace sanitation control and the health management of employees. It would be difficult to achieve the containment of COVID‐19 without full cooperation from the business community. We appreciate your continued support and collaboration.

## 3 SUMMARY TO DATE

In December 2019, cases of severe pneumonia of unknown etiology were reported in Wuhan, China. A new type of coronavirus was detected in these patients, and the WHO named the novel coronavirus disease COVID‐19 on February 11, 2020. The disease spread rapidly throughout the world. On March 11, WHO declared COVID‐19 a pandemic. The novel coronavirus was given the official name SARS‐CoV‐2. Globally, the number of COVID‐19 cases has reached 72 million, with over 1.6 million deaths (as of December 14, 2020). In Japan, the first wave of COVID‐19 spread began at the end of March, but the number of cases started to decline in May, and the state of emergency was lifted on May 25, 2020. The second wave of the pandemic started at the end of July and peaked in August, followed by a decline. During this period, restrictions on the movement of people were relaxed, as shown by the Go To Travel campaign and resumption of cross‐border travel. Since November, there has been a sharp increase in the number of cases, and this is considered the third wave. We are now faced with the challenge of navigating into the upcoming winter months by preventing the spread of COVID‐19 while maintaining economic activities.

### 3.1 What is COVID‐19?

1. Incubation period

The incubation period is thought to last up to 14 days, ranging from 2 to 14 days (median 5‐6 days).

2. Basic reproduction number and effective reproduction number

The basic reproduction number (average number of people infected by a single infectious person in a population with no immunity against the disease) is estimated to be 2.0‐2.5, slightly higher than influenza. Public health interventions (eg, minimizing opportunities to contact with others) can reduce the number of people infected by one patient. This is called the effective production number. Although the effective production number was 1 or less in May 2020, it exceeded 1 in June. As of December 12, the effective production number in Japan was 1.08. When the number of new patients starts to decrease, the effective reproduction number drops below 1, leading to containment of the pandemic.

3. Routes of transmission

Since COVID‐19 is considered to spread through droplets and direct contact with virus contaminated surface of goods, basic infection control precautions should be taken, such as wearing a mask and washing hands. Additionally, microdroplet transmission may also occur in some cases. In this mode of transmission, virus‐containing particles of less than 5 μm linger in the air, traveling distances to infect a person. The WHO and the US CDC call it airborne transmission instead of microdroplet transmission, but the definition differs slightly depending on the institution.

4. Changes in infectiousness

There is a report that the infectiousness of COVID‐19 starts 2 days before the onset of symptoms, peaks immediately after the onset, and rapidly decreases 8 days after the onset. According to another report, a person with a positive PCR test result can have a negative viral culture 7 days or more after the onset. These reports suggest that the infectiousness of COVID‐19 almost disappears in about 1 week after the onset.

5. Testing methods

If COVID‐19 case is suspected by a physician, a nucleic acid test such as PCR, or an antigen test will be performed. Although the antigen test is slightly less sensitive than the nucleic acid test, it is widely used due to its ability to provide results fast. However, quantitative and qualitative tests have some differences in terms of eligible patients and sample locations; therefore, caution is needed when implementing these tests. In addition, there have been reports of false‐positive results with antigen qualitative tests, and the Japanese Association for Infectious Diseases has published the results of a questionnaire survey on false positive results from antigen tests. The US FDA also issued a warning on November 3. Although antibody test kits have been available in clinical settings, it takes more than 1 week from the onset of symptoms before antibodies become detectable in the blood; therefore, they cannot be used for acute diagnosis.^1^, ^2^ Even if you test positive for antibodies, how long the antibodies can last and the ability of the antibodies to prevent infection are often unclear. At this time, we do not recommend personal use of antibody test kits just to feel at ease. As new findings on SARS‐CoV‐2 testing methods are constantly being reported, changes may still occur in the testing methods and interpretation of test results. Comparison of testing methods is presented in Table 1.

6. Vaccines and treatment drugs

**TABLE 1:** Comparison of SARS‐CoV‐2 testing methods

Sample	Nucleic acid test (PCR/LAMP)			Antigen test (quantitative)			Antigen test (qualitative)			Antibody test
	Nasopharynx	Saliva	Nasal cavity	Nasopharynx	Saliva	Nasal cavity	Nasopharynx	Saliva	Nasal cavity	Blood
Purpose of the test	Identification of current infection									Identification of past infection
Eligible persons										
Symptomatic persons										
Within 9 d after onset^a^	○	○	○	○	○	○	○^b^	×^c^	○^b^	Positive for antibodies 1‐3 wk after the onset of symptoms
10 d or more after onset^a^	○	–^e^	○	○	–^e^	○	△^d^	×^c^	△^d^	
Asymptomatic persons	○	○	–^e^	○	○	–^e^	–^e^	×^c^	–^e^	
Pros	High sensitivity (*about 90%*)			Results are ready in about 30 min, but a dedicated device is required			Results are ready in about 40 min by using simple kits			Can be used for epidemiological studies
Cons	Takes half a day to deliver results			Slightly less sensitive than PCR			Less sensitive than PCR. *False positives are reported relatively often*			Difficult to assess during the early phase of infection

^a^ The day of onset is counted as the 1st day.

^b^ An antigen test (qualitative) should be performed within 9 d from the second day of onset.

^c^ The use in symptomatic patients is under investigation, and the use in asymptomatic patients is scheduled to be investigated.

^d^ Available but those who tested negative are subjected to nasopharyngeal PCR.

^e^ Not recommended.

Vaccines are under development in various countries including Japan. Depending on the country, some vaccines have already been approved and started to be administered. In addition, several medications have been tested as candidate treatment drugs for COVID‐19. Remdesivir (in May) and then dexamethasone (in July) as treatment drugs for COVID‐19 are approved by Japanese government.

### 3.2 Stages of pandemic

The Subcommittee on Novel Coronavirus Disease Control has classified the severity of the COVID‐19 pandemic into four stages (Stages 1‐4) and shown response measures for each stage, proposing that all severity levels be appropriately responded to by the central and local governments (August 7, 2020) (Table 2). On December 11, the pandemic severity in regions falling under “Stage 3” was further divided into three levels (continuing to spread, peaking, and declining), and specific measures to be taken by the central and local governments were compiled for each level. Note that the government may declare a state of emergency for regions that reach Stage 4.

**TABLE 2:** Indicators for pandemic severity compiled by the government subcommittee

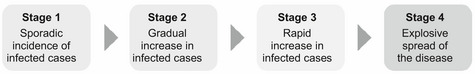							
	Burdens on the medical service system, etc.			Monitoring	Infection status		
	Number of beds occupied		Number of patients per 100 000 people	Rate of PCR positive	Number of new cases reported per 100 000 people	Comparison of the most recent week with the week before	Percent of cases with unknown transmission route
	Total	Severe cases					
Indicators for Stage 3	The occupancy rate of the maximum number of beds secured ≥20%, or the occupancy rate of the number of beds currently secured ≥25%		15 or more	10%	15 or more (1 wk)	Increase over the week before	50%
Indicators for Stage 4	The occupancy rate of the maximum number of beds secured ≥50%		25 or more		25 or more (1 wk)		

## 4 ROLES OF OCCUPATIONAL HEALTH PROFESSIONALS

Occupational health professionals (occupational physicians, occupational health nurse, etc.) are assigned to a workplace with 50 or more employees. In order for business operators to make appropriate decisions in infection risk management, occupational health professionals are expected to provide active support as health experts in their workplace while collaborating with health supervisors. When developing response measures, it is necessary to keep in mind that each workplace has different working conditions.

### 4.1 Major roles of occupational health professionals

1. Collecting medical information and providing it to the workplace
  - Collect the latest information from the central and local governments and various agencies in Japan and overseas and provide it to the employer and workers.
2. Assessing and advising on the medical validity of infection prevention measures
  - Assess the medical effectiveness and feasibility of control measures and advise on and coordinate the actions for risk management.
3. Assessing and coordinating education/training on infection prevention and control
  - Provide education/training and implement infection prevention activities and appropriate infection control.
4. Considering and implementing measures accommodating employees’ health conditions
  - Identify employees with underlying diseases and other high‐risk workers and plan necessary accommodations in advance.
5. Responding to the situation where a worker has been identified as a COVID‐19 case (including a suspected case)
  - Advise on and coordinate the response to the infected worker and the prevention of secondary infection to avoid a workplace cluster.
6. Giving consideration to employees’ mental health
  - Help alleviate the anxiety and stress among employees by providing appropriate information.
7. Considering and advising on the medical validity of phased de‐escalation or escalation of restrictions
  - Advise on and coordinate measures for infection control in the process of de‐escalation or escalation of restrictions.
8. Assessing and advising on the medical validity of medium‐ to long‐term measures
  - Advise on and coordinate actions aimed at permanent infection prevention for supporting the company's business continuation without increasing infection risks among workers and in the workplace.

## 5 MEASURES IN THE WORKPLACE

### 5.1 Overview

As a "safety assurance" for employees, employers are advised to actively adopt telework to prevent employees from contracting the disease during commuting and in the workplace. Also from the viewpoint of "social responsibility," they must strive to reduce contact opportunities among employees in response to requests for voluntary restrictions on movement/outings from the central and local governments. Furthermore, employers are required to implement infection prevention measures and behavior modification to avoid a workplace cluster, for which they need to provide accurate information and explanations about the policies for employees and their families. As the disease spreads further, some employees will be forced to stay home due to a family member needing care, school closure, etc. Therefore, appropriate personnel policies should be put in place by referring to the “Q & A for employers”, etc.

### 5.2 Risk communication

During the epidemic of a new pathogen, risk communication can be a challenge due to a lack of information. The US CDC provides six principles of emergency risk communication in an infectious disease outbreak (Table 3). WHO also provides guidance on risk communication regarding COVID‐19 on its website.

**TABLE 3:** Six principles of emergency risk communication during a disease outbreak (US CDC)

Be First (share quickly)	In addition to communicating information, “who” communicates the information is important
Be Right (be accurate)	Communicate both "what is known" and "what is not known"
Be Credible (gain trust)	"Scientifically based information" heightens the trust of the recipient
Express Empathy (be compassionate)	Communicate information from the standpoint of the recipient
Promote Action (support action)	Emphasize that the behavior of each person leads to infection prevention
Show Respect (respect others)	Try to convey information in a manner that respects the other person's position and rights

Build a platform that enables information distribution and sharing at the right time (Table 4). When sharing information about an infection case in the workplace, consideration needs to be given to not just the disease information but also the worker's privacy. Therefore, it is necessary to specify the amount and type of information to be disclosed and the procedure in advance.

**TABLE 4:** Examples of the platforms to connect employers and their employees

Intranet (special page)	The amount of information is adjustable, but it is prone to be one‐way communication.
Internal SNS (chat tool)	Real‐time information sharing is possible regardless of the location.
Internal Wiki	Shared information can easily be edited and updated.
Bulletin board (paper medium)	An important source of information for employees not using computers

### 5.3 Basics of infection prevention

Important points in the prevention of workplace transmission are shown in 1. to 3. below. The checklists provided by the Ministry of Health, Labour and Welfare (MHLW) and ILO (International Labour Organization) are useful for checking the implementation status of infection control in your workplace. Universal wearing of face masks, hand hygiene practice, and avoidance of the Three Cs (closed spaces, crowded places, and close‐contact settings) are the basics of infection prevention.

1. Wearing a mask (universal masking)
  - COVID‐19 is most contagious 2 days before the onset of symptoms until immediately after the onset, and a certain proportion of infected people remain asymptomatic. Asymptomatic carriers may spread the disease through virus‐containing droplets released while speaking, etc. in public places, “wearing mask by everyone including persons with no symptoms” is recommended. This effort is called universal masking (or universal mask).
2. Practice of hand hygiene
  - Hand hygiene should be practiced basically by washing hands to prevent contact transmission. It is also important to avoid touching the face and eyes. The principle of handwashing is to wash off virus adhering to the surface of the hands and fingers with soap and water. If soap and water are not available, use alcohol‐based disinfectant solution (60%‐95%).
  - In general, the concentration of alcohol in disinfectant solutions is by volume. In the Fire Service Act, the concentration of alcohol in a disinfectant classified as a hazardous material is 60% or more by weight and is thereby different from the concentration expressed as volume percent. Therefore, caution is needed.
3. Avoidance of the "Three Cs"
  - Implement measures (environmental arrangement and restriction of movement) to avoid the “Three Cs.” Even if the “Three Cs” do not overlap, try to achieve "Zero C" as much as feasible to reduce cluster risks.
  - Avoid "5 situations" that increase the risk of infection not only during, but outside working hours as well (including lunch break). It is important to provide workers with certain guidance on infection prevention behaviors outside working hours.

### 5.4 Development of systems and procedures

1. Social responsibility (CSR)
  - Business operators must recognize their social responsibility (CSR), which goes beyond preventing the spread of the disease in their workplaces and related sites, and strive to contribute to the infection control efforts made by their clients, business partners, industry organizations, and communities through active provision/sharing of information.
2. Confirmation of medical validity
  - Before establishing a system or procedure, seek advice from the occupational health staff as to whether it is medically appropriate. Industry‐specific guidelines are available from MHLW and other relevant ministries and agencies for reference.
3. Wage support
  - Uninfected employees may still need to take leave of absence if a family member or someone living in the same household has become infected and needs care. For that reason, support should be provided to cover lost wages during their leave.
4. Prevention of discrimination
  - The majority of infected cases occur unexpectedly. To ensure that there will be no discrimination against people with confirmed or suspected COVID‐19 and those identified as close contacts, the company needs to provide education for employees and their families.
5. Support for employees who work from home
  - For employees who telework, support measures are needed to prevent mental health problems due to lack of communication or health problems due to lack of exercise. Providing detailed support is particularity important for newly hired employees who start teleworking immediately after joining the company.
6. Adoption of COVID‐19 Contact‐Confirming Application (COCOA)
  - COVID‐19 Contact‐Confirming Application (COCOA) enables the users to receive notification of possible exposure to a person infected with COVID‐19 while ensuring the protection of privacy. Make sure that infected employees register themselves by providing accurate information.
  - Prepare response measures and a protocol for the situation if a employee has received a notification of possible exposure to COVID‐19 case.

### 5.5 Environmental control

#### 5.5.1 Ventilation in the workplace

During the winter months, it is important to ventilate the room air frequently while preventing a temperature drop. There are roughly two methods of ventilation that are recommended: (i) opening windows and (ii) mechanical ventilation (the maintenance of air conditioning/ventilation systems). In either method, make sure to maintain an adequate humidity level (40% or higher). As a rough guide for proper ventilation, you can measure the concentration of carbon dioxide in the room to make sure that it does not exceed 1000 ppm.

1. By opening windows
  - Natural ventilation through opened windows while maintaining an adequate room temperature and humidity is an effective method. Ventilation should be performed at least once every 30 minutes, preferably by leaving the window fully open for a few minutes. To create a cross flow, a window at the opposite side should be opened as well (if the room has multiple windows). If there is only one window, the door should be kept open.
2. By mechanical ventilation
  - Check whether the air quality criteria and the required ventilation rate (30 m^3^/hour/person) are satisfied. If the required ventilation rate is not satisfied, clean or perform proper maintenance on the ventilation system.
  - For air conditioning, the ventilation rate should be maintained at all times, and make sure that workers do not adjust the flow rate without permission.
  - To check whether the required ventilation rate is satisfied, a free ventilation simulator is available from the JSOH (Expert Community of Occupational Hygiene & Ergonomics).

#### 5.5.2 Cleaning and disinfecting workplace

Contact transmission occurs when the mucous membranes of the mouth, nose, and eyes are touched with the hands/fingers contaminated with the virus. In the workplace, there are items used by individual workers as well as machines and equipment touched by multiple workers. Therefore, performing routine environmental disinfection can help prevent transmission of the virus (Table 5). When using personal protective equipment (PPE), it is also necessary to provide employees with education/guidance on how to put on and take off the PPEs and how to dispose of contaminants. Although there are products on the market claiming to have high "disinfecting or sterilization" effects, it is important to thoroughly examine their effectiveness under actual use conditions and select a product suitable for the purpose.

1. Regular environmental disinfection
  - Routinely disinfect high touch surfaces such as doorknobs, handrails, and elevator buttons.
  - Routinely disinfect toilets (including the floor) as they are shared by many.
  - Disinfection should be performed at least once a day (preferably more than once).
  - It is desirable to disinfect the desk, chair, PC, phone, etc. by wiping down with alcohol at the end of each workday (before and after use if they are shared).
2. Disinfection after a worker has become infected
  - The employer is responsible for carrying out disinfection of the workplace in accordance with the instructions given by a public health center.
  - If no instruction has been by the public health center, perform disinfection by using the following as a guide:
    - In principle, all areas/surfaces used by the infected person less than 3 days ago are subjected to disinfection.
    - Ensure adequate ventilation before disinfection. The US CDC recommends ventilating the area for about 24 hours before disinfection.
    - Areas to be covered by disinfection include the infected person's work area, conference rooms (desks, chairs, etc., within a radius of about 2 m), and restrooms, smoking room, refreshment room, cafeteria, etc. used by the infected person.

**TABLE 5:** Basic concept of workplace disinfection

Clean surfaces using a neutral detergent prior to disinfection Use alcohol‐based disinfectant solution (60%‐95%) or *sodium hypochlorite (0.05%)* To disinfect toilets, use *sodium hypochlorite (0.1%)* Disinfection should be done basically by wiping. Use of a disinfectant spray in a space should be avoided as it may cause inhalation Use appropriate personal protective equipment (mask, protective glasses, gloves, gown, etc.) where necessary *Hypochlorous acid water* is *thought to be effective* for disinfection against the novel coronavirus depending on the available chlorine concentration and method of use. However, many of these products have shown no clear evidence of effectiveness or safety and therefore should be avoided As for products claiming to be *space disinfectants*, *no rational basis for their effects has been confirmed*

#### 5.5.3 Management of an onsite clinic

1. Operation of the clinic
  - An onsite clinic may cause the spread of the disease if visited by many employees with unwell. Therefore, give thorough consideration to how the clinic should be operated, including measures such as limiting services or shutting it down.
  - If continuing to provide medical services, the use of remote consultations via phone or the Internet and mail‐delivery prescriptions should be recommended. When implementing these measures, take into consideration the important points to note, etc.
2. Implementation of infection prevention measures
  - Healthcare professionals in onsite clinics and medical check‐up sites should follow the standard precautions and implement appropriate infection prevention measures (mandating mask wearing, separating the waiting line and the flow line, ensuring a safe physical distance, securing a waiting area for those with unwell, etc.)
  - When using an N95 mask, conduct a fit test beforehand. For information about medical protective equipment such as fit‐testing and reuse of N95 masks, please refer to the website of the Research Group of Occupational Infection Control and Prevention in Japan.

### 5.6 Behavior modification of employees

As the COVID‐19 pandemic may continue for months to come, response measures should be developed by taking into consideration the possibility that further “reinforcement of behavior modification” may be requested.

#### 5.6.1 Physical distancing

Preventing the spread of the disease mainly by maintaining a physical distance, such as avoiding crowded places and securing a safe distance from other people, is called physical distancing (or social distancing). In the workplace, physical distancing should be practiced by implementing the following measures:

1. Personnel placement in work spaces and offices
  - Arrange seats in such a way that a distance of at least 2 m can be maintained between people during work.
  - Limit face‐to‐face business activities (including meetings and training) and actively use Web conferences, etc.
  - Minimize the number of visitors from outside the company (eg, guests, parcel delivery, mail delivery, and lunch delivery).
  - Limit the number of people allowed in an elevator at one time and avoid engaging in conversations while riding the elevator as much as possible.
2. Management of the cafeteria, refreshment room, dress changing room and smoking room
  - To avoid the "Three Cs" in the cafeteria, limit the number of people allowed in at one time and the use time, install partitions such as acrylic boards on the tables, refrain from talking while eating, try to maintain a distance of at least 2 m between people, and consider shutting down the cafeteria if necessary. It is also recommended that employees bring a lunch to eat at their own desk quietly.
  - In a place such as a refreshment room and dress changing room, people tend to remove their masks and talk with each other due to the switch of environment, which may increase the risk of virus transmission.
  - In a smoking room, masks are removed and the conditions for the "Three Cs" are satisfied. Therefore, it is advisable to shut down the smoking room. If shutting down is not feasible, avoid the "Three Cs" by limiting the number of people allowed in at one time.
3. Management of the flow of people in work spaces and offices
  - Fix and limit the flow of people (eg, restrict work areas and floors, one‐way traffic patterns, separate entrance and exit at each building/office) to minimize contact opportunities. Fixing and limiting the flow of people is an effective measure for contact tracing when someone in the workplace has become infected with the disease.
  - Divide members in a single department into two groups and have Group A work in the office while Group B work from home. This allows the continuation of business operations even when a member of the department has been infected with COVID‐19, which would require all members of the department to quarantine as close contacts if they worked together in the office.

#### 5.6.2 Management of free‐address workspace

In the free‐address work style, employees have no assigned desks and are free to choose where to sit. In a free‐address workspace, it is easy to secure a safe distance between people. However, if anyone has become infected, identifying the close contacts may become a challenge. For that reason, the following points should be noted when continuing with free‐address working:

1. Recording and tracing of activity history
  - Employees should keep a record of the desks they have used and places they have stopped by (record the activity history) to make it easy to identify close contacts if a COVID‐19 case arises.
  - To improve the accuracy of contact tracing, it is effective to limit workspaces for employees (floors and areas) as much as practical.
2. Disinfection of machines and equipment
  - It is desirable that employees disinfect shared machines and equipment, such as desks, chairs, and phones, by wiping down with alcohol before and after use.
  - Place adequate amounts of alcohol disinfectant solution, disposable paper towels, and trash cans in the workplace.

#### 5.6.3 Health measures for employees who work from home

More than a few employees are likely to find it difficult to maintain the sense of belonging and feeling of security due to the prolongation of telework. There is also no denying that some employees experience "the feeling of loneliness" or "difficulty working," although its extent varies among individuals. To ensure smooth teleworking experiences, measures are needed for intangible aspects in addition to the enhancement of tangible aspects. Table 6 shows some of the challenges (pros and cons) that can be experienced when teleworking. For legal considerations associated with telework, refer to “Examination of legal considerations of telework” in this Guide.

**TABLE 6:** Challenges of teleworking

	Employee	Business operator
Pros	Improves work‐life balance Reduces the time‐related and physical burdens without commuting. Easier to focus on work (Familiar devices and equipment) Possible to balance work and childcare/nursing care	Reduces infection risks among employees Improved labor productivity can be expected Reduces office‐related costs Reduces interpersonal problems
Cons	Difficult to distinguish between work and private life May reduce the sense of belonging Difficult to stay motivated Easy to get out of shape May induce mental health problems, such as feelings of isolation, difficulty adapting to change, and day‐night reversal. Difficult to secure a non‐distracting environment (Inadequate chair, desk, lighting, noise control, etc.)	Difficult personnel management Likely to diminish two‐way communication May increase the risk of information leakage Difficult to conduct education/training and performance evaluation May reduce work productivity

To ease these challenges, consider taking the following measures to create better teleworking environments:

1. Separate your professional and private life.
  - Notify your supervisor at the time of starting and ending work.
  - Step outside the house during a lunch break for strolling, etc.
  - Get dressed and look presentable each morning, just like you would when going to the office.
  - Avoid work‐related emailing and other type of communication outside the working hours except in case of emergency.
2. Consideration of the methods of communication
  - Active communication via teleconferencing and web conferencing tools is recommended.
  - Regularly hold meetings at set times through audio and screen sharing.
3. Understand the limitations of teleworking.
  - Share the pros of teleworking with others while keeping in mind its cons.
  - Since it is impossible for a company to arrange appropriate work environments for individual employees, they should not be expected to produce the same level of work output as in the office.
  - Constantly seek ways to increase productivity during teleworking among teams and implement them.

#### 5.6.4 Measures for face‐to‐face service operations involving a large number of people

In jobs requiring interactions with a large number of people (eg counter services and sales operations), it is recommended to take the following measures in addition to the basic precautions (mask wearing, hand hygiene practice, and avoidance of the Three Cs).

- Install non‐flammable transparent sheets and acrylic boards to prevent respiratory droplets from spreading.
- If necessary, wear a face shield to prevent transmission through the eyes.
- Make sure to thoroughly ventilate the space, avoid talking loudly, and secure a safe physical distance (try to maintain a distance of 2 m between people while waiting in line at a store, etc.).

#### 5.6.5 Challenges associated with business trips

The risk of infection associated with a business trip depends on the mode of transportation (airplane, train, etc.) and the pandemic severity in the country/region. Therefore, when assigning an employee to a business trip, the necessity of the trip and the risk of infection should be fully assessed. In some areas in Japan, the number of COVID‐19 cases has shifted to an increasing trend, and it is advisable to carefully examine the necessity of the business trips across high‐risk prefectures. With regard to overseas business trips, phased relaxation of travel restrictions has been underway for workplaces. Therefore, when assigning an employee to an overseas business trip, the necessity of the trip and infection risk should be thoroughly evaluated, as with a domestic business trip. For details of the handling of overseas business travelers and expatriates, refer to Chapter 4 "Measures for Overseas Travelers and Expatriates" in this Guide.

#### 5.6.6 Other challenges

1. Securing an opportunity for employee health checkups
  - It may become difficult for employees to receive health checkups at a designated medical institution due to the spread of COVID‐19. Consider allowing the use of other institutions (by voluntary submission of checkup results) so that they can still have an opportunity for health screening.
  - As for special health examinations (for those exposed to a harmful factor), it is necessary to provide the doctor conducting the examination with information about the working environment and procedures in advance to help with a brief survey on working conditions and medical interview.
2. Securing necessary equipment in the workplace
  - Stock up on necessary supplies and secure purchasing routes based on past shortages of personal protective equipment (masks, gowns, etc.), alcohol disinfectant solution, etc.
3. Considerations for addiction
  - Attention should be paid to concerns about potential worsening of addiction, such as alcohol dependence and gaming disorder, due to anxiety and stress over uncertainty.
4. Recommendation of smoking cessation
  - Smoking cessation is strongly recommended as smoking is considered a risk factor for severe symptoms. It is advisable to promote cessation support for smokers in the workplace.

### 5.7 Infection prevention by employees

#### 5.7.1 Infection prevention efforts personally carried out by employees

1. Health monitoring
  - Conduct daily health observation (eg body temperature and physical condition check) and stay home when having fever or cold symptoms.
  - Stay home when feeling unwell, even without a fever. If an employee becomes sick at work, immediately send them home.
2. Hand hygiene practice and mask wearing (Table 7)
  - Take basic precautions to prevent infection, such as hand hygiene practice and mask wearing. It is also important to avoid touching the face and eyes.
  - The principle of handwashing is to wash off virus adhering to the surfaces of the hands and fingers with soap and water. If soap and water are not available, use alcohol‐based disinfectant solution (60%‐95%).

**TABLE 7:** Points to note when wearing a face mask, etc.

*When wearing a mask while working in the heat* When working in the heat or performing high‐intensity work with a mask on, *give consideration to* (1) shortening the duration of continuous work and (2) removing the mask to take a break in a cool place where a sufficient distance can be kept from other people. *Proper use of face shields and mouth shields* The intended use of the face shield is to prevent transmission of the virus through the ocular mucosa by respiratory droplets. The use of a face shield alone is *expected to have little effect on reducing* the amount of respiratory droplets released or inhaled. Therefore, a face shield should always be used with a mask for infection prevention. A mouth shield is also *expected to have little effect on reducing* the amount of respiratory droplets released or inhaled and should not be used for the purpose of infection prevention. When using a face shield or mouse shield to show lip movements (such as for sign language), maintain an appropriate physical distance to prevent transmission.

#### 5.7.2 Response to employees with unwell such as fever and cold‐like symptoms

There have been many reports of COVID‐19 cases exhibiting almost no symptoms (with slight fever or no fever). Employees presenting with fever or cold‐like symptoms should be encouraged to consult with “their primary physician/the nearest medical institution” or “the COVID‐19 medical consultation service” provided by the local government and get tested for the disease. Even after the fever or cold symptoms improve, the possibility of infection cannot entirely be ruled out if the employee did not visit a medical institution or did visit a medical institution but did not receive SARS‐CoV‐2 testing. In such case, it is recommended to follow a return to work procedure based on the general guidelines in Table 8. If an employee tests negative for COVID‐19 after the onset of symptoms and COVID‐19 infection is considered unlikely due to other symptoms, confirm that at least 72 hours have passed since the resolution of the fever and cold symptoms before the employee is allowed to return.

**TABLE 8:** General return to work guidelines for employees who have not been tested for COVID‐19

They may return to the workplace when all of the following conditions are met: At least 8 d have passed since onset. At least 72 h have passed since the resolution of fever^b^ and other symptoms^a^ are improving If it is difficult to be absent from work during the above period, the employee should try to get tested for COVID‐19 as much as possible. If that is not possible, the following measure may be taken under the responsibility of the company: Confirm that at least 72 h have passed since the resolution of fever and cold‐like symptoms^b^ before allowing the employee to return.
Requesting certificates ("negative result or recovery certificate") should be avoided as much as possible as these increase the workload of medical institution, etc. After returning to the workplace, the employee should continue to take the basic precautions, such as daily health observation, mask wearing, and physical distancing. These guidelines may not apply to telework, but caution should be exercised to avoid household transmission.

^a^ Not taking medications for symptomatic relief, including a fever reducer.

^b^ Such as cough, malaise, and respiratory discomfort.

For a return to work procedure for employees who have contracted COVID‐19, refer to “Return to work guidelines for infected employees” in this Guide.

#### 5.7.3 If an employee is infected

In principle, infected employees and those identified as close contacts should be managed according to the instructions from a public health center, etc. However, in an area experiencing a growing number of COVID‐19 cases, it may take time until specific instructions are provided by the local public health center. Therefore, it is advisable that business operators prepare a protocol that can facilitate prompt responses without waiting for instructions from the health center. The physician who has diagnosed the employee with COVID‐19 is required to notify the designated public health center in the area according to the Infectious Diseases Control Law. Usually, the notified public health center contacts the patient's place of work, which initiates contact tracing to identify close contacts. In reality, a report from the infected employee to the company comes first. Therefore, it is desirable that the employer contact the local public health center upon receipt of the report and ask for instructions (without waiting for the center to contact first).

1. Prohibition of discrimination
  - There must be no discrimination or bias against not only infected workers but also their family members and colleagues.
  - Ensure that all persons concerned understand that no harassment, bullying, pushing for resignation, and slandering on social networking sites based on uncertain information will be tolerated.
2. Cooperation with a public health center
  - Appoint someone to act as a liaison with a public health center beforehand.
  - Prepare the floor plan of the department where the infected person works (desk arrangement, desk size, width of aisles, etc.) and a record of anyone who came in contact with the infected person inside and outside the workplace (meetings, lunch, dinner, etc. 2 days before onset or later).
  - Perform workplace disinfection under the responsibility of the employer. If necessary, consult with the public health center to decide whether to temporarily close off the work area or part of the office where the infected worker has been present. However, there is no need to consider partial or complete shutdown of the facility every time a worker becomes infected.
3. Start of self‐isolation
  - An employee confirmed as infected should start self‐isolation as per instructions by the public health center and medical institution.
  - Those identified as asymptomatic pathogen carriers or with mild symptoms are instructed to self‐isolate at hotel or home without hospitalization.
  - If either a hotel or home can be selected as a place of self‐isolation, it is desirable that the employee be aware that staying in a hotel is recommended.
    - In a hotel, appropriate action can be taken at the right time if a sudden change occurs in symptoms. Furthermore, the risk of household infection can be avoided.
    - In principle, persons living with the infected person are considered close contacts. For that reason, when an infected person self‐isolates at home, those residing in the same household may be placed in health observation for an additional 14 days from the day when the self‐isolation ends for the infected person.
4. Return of the infected employee to the workplace
  - Infected people are most contagious 2 days before onset till immediately after onset,^3^, ^4^, ^5^ and the infectiousness is considered to disappear almost completely by about 1 week after onset.^3^
  - Regarding the removal of the restrictions on attendance at work based on Article 18 of the Infectious Diseases Control Law, PCR testing before hospital discharge is not mandatory, and the restrictions may be lifted “if 10 days have passed since the onset and 72 hours have passed since the resolution of symptoms.” General return to work guidelines for infected workers are summarized in Table 9.

**TABLE 9:** General return to work guidelines for infected workers

They may return to the workplace when all of the following conditions are met: At least 10 d have passed since the onset (or after a definitive diagnosis). At least 72 h have passed since the resolution of fever^a^ and other symptoms^b^ are improving.
Seek advice from the attending physician, occupational physician, etc. and return to work in a reasonable manner. Requesting certificates (*"negative result or recovery certificate"*) should be avoided as much as possible as these increase the workload of medical institutions, etc. After returning to the workplace, the worker should continue to take the basic precautions, such as daily health observation, mask wearing, and physical distancing.

^a^ Not taking medications for symptomatic relief, including a fever reducer.

^b^ Such as cough, malaise, and respiratory discomfort. (However, taste and smell disorders may be prolonged).

#### 5.7.4 If a employee has been identified as a close contact

1. If a employee has been identified as a has been identified as a close contact based on an active epidemiological investigation conducted by a public health center, implement infection prevention measures by following the instructions from the designated public health center in the area.
2. The employer should provide the public health center with information on the employee (the name, age, address, telephone number, workplace seating chart, activity history, anyone who has come in contact with the employee during meetings and dining, etc.).
3. In areas where COVID‐19 cases are growing, it may take time for the public health center to provide instructions. Therefore, persons suspected as close contacts may be placed in home quarantine at the discretion of the employer.
4. PCR testing (initial screening) is performed for all close contacts. Even persons who tested negative are instructed to be placed in 14‐day health observation from the date of the exposure to the “patient (confirmed case)” within the infectious period.
5. In principle, persons living with the infected person are considered close contacts. For that reason, when a family member of a employee residing in the same household has contracted COVID‐19 case and is going to self‐isolate at home, the employee may need to be placed in health observation for an additional 14 days from the day when the self‐isolation period ends for the infected person.
6. If employers decide on their own to instruct close contacts or persons not considered close contacts to quarantine at home or extend the health observation period, the actions must be supported by relevant laws, employment rules, etc.
7. If a employee who was not considered a close contact by a public health center wishes to get tested to be reassured, the employee may do so at a medical institution where SARS‐CoV‐2 testing is available, basically at their own expense (Table 10).

**TABLE 10:** Definition of a close contact

A “close contact” refers to a person who meets certain conditions among those who came in contact with a “patient (confirmed case)” during the infectious period. This includes persons who live with or had prolonged contact with the patient (confirmed case) and those who were within a distance close enough to touch by hand (about 1 m) from the patient (confirmed case) for 15 min or more without taking necessary precautions. In principle, PCR testing is performed for all “close contacts” For details, see the *Guidelines for Active Epidemiological Investigation in Patients with Novel Coronavirus Infection*

#### 5.7.5 Accommodations for high‐risk persons

For high‐risk persons (those who have risk factors for severe symptoms), provide appropriate accommodations to prevent virus transmission. Usually, high‐risk persons are identified based on the results of health checkups, but these provide only limited information. Therefore, set up an environment enabling high‐risk persons to self‐report. In doing so, establish an appropriate procedure to obtain health information from such employees while protecting their privacy. Explain to the employees that their information will be strictly managed by the occupational health staff or health supervisor and used only for the purpose of infection prevention in the workplace. Risk factors for severe symptoms are shown in Table 11.

**TABLE 11:** Risk factors for severe symptoms

Confirmed risk factors for severe symptoms	Important underlying disease under evaluation
Elderly patients aged 65 y and older	
Chronic obstructive pulmonary disease (COPD)	Use of biologics
Chronic kidney disease	Post‐transplant or other immunodeficiency
Diabetes mellitus	HIV infection (particularly CD4 <200/μL)
Hypertension	Smoking history
Cardiovascular disease	Being pregnant
Obesity (BMI ≥30)	Malignant tumor

The following are specific examples of work accommodations (Table 12):

**TABLE 12:** Examples of work accommodations

Provide accommodations to reduce the risk of infection (adjustments to starting time, commuting by car, etc.) Allow telework or self‐isolation at home to reduce the risk of contracting the disease by coming to work For employees undergoing outpatient treatment, make necessary arrangements so that they can continue with the treatment Provide opportunities for face‐to‐face consultations with occupational physicians and public health nurses when desired. Avoid assigning jobs that require contact with a large number of people Ensure adequate ventilation in their work space (by the air‐conditioning unit or windows) Avoid business trips (both domestic and overseas) to regions where COVID‐19 cases are growing Improve the paid leave system by utilizing *the leave allowance subsidy*, a maternal health care measure implemented in response to COVID‐19

#### 5.7.6 Other matters to consider

- Since employee education is most essential in the prevention of COVID‐19 transmission and takes high priority, it should be implemented through collaboration between employees and management.
- Educate employees about transmission routes, factors contributing to cluster development, initial symptoms, preventive methods, and response to a COVID‐19 case in the workplace. Have them perform daily health management, check their body temperature before coming to the office each day, and record daily activities.
- Supervisors need to create a climate where subordinates can feel free to approach them and ask for advice. Make sure that employees should understand that it may cause the spread of the disease when they, despite feeling sick, come to workplace not to make any inconvenience of their supervisors or colleagues.
- Since infected employees deal with anxiety, provide them and their families with psychiatric support, as well as wage support during leave (including self‐quarantine).
- Employees may feel excessive anxiety associated with COVID‐19 (eg, I may have become infected, I can't help watching COVID‐19‐related news all the time, I feel stressed in my daily life, etc.), which can interfere with their day‐to‐day living. If you notice a employee having such anxiety, seek advice from the occupational health staff, etc. as to how to support the employee.
- A employee who has taken leave due to a mental health problem may submit a medical certificate issued by the employee's primary physician stating that “the employee is able to work again by means of teleworking.” Implementation of telework may entail labor management issues and stress unique to working from home, and it does not necessarily reduce the employee's workload. With the consent of the employee, the occupational health physician should carefully examine whether a telework arrangement is appropriate for the employee based on the information and opinions obtained from the employee's primary physician.

#### 5.7.7 Proof of negative SARS‐CoV‐2 test in workplaces

There have been more and more situations where the submission of a negative result certificate is required, such as when participating in an event, going on a domestic business trip, transferring to a new workplace, and being admitted to a facility. Although PCR is the most sensitive testing method for COVID‐19, it also produces certain numbers of "false‐negative" and "false‐positive" results. Also with PCR, it is possible for a person who tested negative to turn positive later; therefore, it does not lead to a complete sense of security. With an understanding of this background, Table 13 summarizes the points to note regarding negative result certificates in workplaces. Please refer to the "Basic Concept and Strategies for COVID‐19 Testing System" in the proposals of the 13th Subcommittee on Novel Coronavirus Disease Control (October 29, 2020).

**TABLE 13:** Points to note regarding negative result certificates

Purpose	Testing required	Remarks
Business travel	○	An agreement between governments. Mostly PCR is required prior to departure. For details of the testing methods, check with the embassy of the destination country in Japan
Recovery certificate	×	There is little medical validity to the issuance of a negative result certificate as the infectiousness decreases rapidly about 1 wk after the virus transmission. Requesting certificates that can increase the workload of medical institutions should be avoided as much as possible
Other	△	Consider the medical validity and significance as social economic activities and make proper judgments based on individual circumstances
Remarks	For asymptomatic persons, the tests are performed at their own expense in a medical institution where the testing is available. Public health centers do not provide the testing for the issuance of negative result certificates If an employer considers providing SARS‐CoV‐2 testing at the workplace clinic, it is necessary to thoroughly discuss the “protocol for positive cases” and “possibilities of false‐negative and false‐positive results”	

## 6 MEASURES FOR OVERSEAS BUSINESS TRAVELERS AND EXPATRIATES

As the COVID‐19 pandemic continues worldwide, it has become necessary to develop health measures for overseas business travelers and expatriates. In doing so, employers have to not only ascertain their own health problems associated with the pandemic but also the Japanese government's travel advice and immigration policies, and border control measures enforced by destination countries/regions.

### 6.1 Impact of COVID‐19 pandemic and health measures

Depending on the severity of the pandemic in the country of stay, the risk of SARS‐CoV‐2 infection may be higher than expected in overseas employees than in domestic employees. Therefore, it is important to make additional careful efforts to prevent infection than in Japan. During disease spread, it should be assumed that the capacity of medical institutions, including emergency services, will be limited and that there will be shortages of medical products such as drugs and vaccines. Take extra precautions in countries/regions with fragile healthcare infrastructure. If a employee experiences symptoms suggestive of COVID‐19 in the country of stay, the employee will be tested and treated according to the healthcare system of the country. Anyone suspected of having COVID‐19 is not allowed to board a commercial flight. Therefore, if the country/region where the employee is staying does not have sufficient medical services, it is necessary to consult a medical transportation company about the potential need for air transport to a neighboring country.

### 6.2 Overseas health problems associated with COVID‐19 pandemic

With the spread of COVID‐19 disease, various health problems can occur in overseas employees. To develop health measures, these health problems need to be understood.

1. Infectious diseases other than COVID‐19
  - The COVID‐19 pandemic has caused delays in medical system operations, which have often led to poor sanitary conditions and decreased vaccination rates including routine immunization, contributing to the spread of other infectious diseases.
  - Currently, there are growing cases of dengue fever in South America, measles in the Philippines and other regions, and tick‐borne encephalitis in European countries. To prepare for these situations, it is recommended that overseas employees receive the necessary vaccinations (including influenza vaccine) before leaving Japan.
2. Lifestyle diseases
  - Long‐term restrictions on outings, lack of exercise due to telework, imbalanced diet, and shortages in regular medications may worsen lifestyle diseases, leading to the development of secondary diseases.
  - Being unable to receive health checkups raises concerns about difficulties in evaluating the health status of expatriates. Additionally, expatriates who regularly take medications for chronic conditions may have difficulty obtaining their medications, thereby requiring arrangements with local medical institutions.
3. Mental health problems
  - Anxiety and fear about COVID‐19 can affect a person's day‐to‐day living, and difficulty making predictions about the future can bring about high intense stress.
  - Post‐entry quarantine and long‐term avoidance of non‐essential activities in a foreign country can lead to a strong sense of isolation and take away opportunities for stress relief. Expatriates are prone to mental health problems due to limited stress relievers and lack of support systems.

### 6.3 Health measures for employees working overseas

1. Obtaining information about the destination
  - Collect important information such as Travel Advice and Warning on Infectious Diseases provided by the Ministry of Foreign Affairs (MOFA) and border control measures.
  - Check information about testing and obtaining a certificate of negative test result and medical institutions that can issue the certificate.
  - Check the severity of the COVID‐19 pandemic in the destination (number of new cases, deaths, etc.).
  - The prevalence of other infectious diseases should also be checked before departure.
2. Establishment of a health support system
  - Make sure that overseas employees (duration of 3 months or longer) are registered with the "Overseas Residential Registration (ORR) net" to establish a network that can facilitate information distribution/gathering for overseas employees. Employees who are staying overseas for less than 3 months should be registered with “Tabi Regi.”
  - Gather and organize information on medical institutions (consultation hours, services, high‐fever consultation, online consultation, health checkups, etc.).
  - Set‐up and promote the use of consultation services for expatriates who may have difficulty obtaining medications for their chronic diseases (hypertension, diabetes, etc.) or those who need health‐related advice due to prolonged restrictions on non‐essential activities.
  - Develop a protocol in case an overseas employee contracts COVID‐19 case (how to seek medical attention, involvement of an assistant company, method of transport, etc.).
  - Check whether the expatriate's medical insurance covers medical costs associated with COVID‐19. For employees going on a business trip, purchase overseas travel insurance that covers COVID‐19‐related medical expenses.
  - Establish a system that enables the provision of necessary supplies such as disinfectants and face masks.
3. Health risk assessment for employees working overseas
  - In addition to expatriates, evaluate the health status of employees assigned to go on business trips to determine if they are sufficiently healthy to endure the trip.
  - Health risk assessment should consider the employee's age, medical history, underlying diseases, immunization history, lifestyle (smoking), and past health records, as well as the epidemic situation in the destination, MOFA’s travel advice and warnings, entry restrictions enforced by the country of stay, etc.
  - In principle, high‐risk persons with chronic diseases should be excluded from candidates for business trips and global assignments to Level 3 regions according to MOFA’s travel advice and warnings. If a employee who has already been relocated overseas is determined to be high‐risk, consider having the employee return to Japan temporarily.
  - For other diseases (infections, lifestyle diseases, mental health problems, etc.), similarly evaluate the employees’ risks during overseas travel and take appropriate measures.
  - As for whether or not to relocate with family, it is advised that individuals make more careful decisions than in pre‐pandemic times based on the local epidemic status, medical infrastructure, MOFA’s travel advice and warnings, etc.
4. Health guidance for employees working overseas
  - When employees are assigned to work in a country/region where entry from Japan is restricted, inform them beforehand that they will be subjected to a 14‐day restriction of movement, including self‐isolation (where they are not allowed to take even a step out of their hotel room).
  - If employees working overseas show symptoms suggestive of infection, have them seek medical attention at a local hospital that accepts patients with fever or a designated medical institution. Also remind them to always carry a means of communication (a cellular phone and charger).
  - Advise them to take at least a 3‐month supply of regular medications with them to ensure that they can continue to receive treatment overseas.
  - Establish a system that enables the conduct of web interviews, etc. in an overseas location.
5. Consideration of evacuation from overseas
  - In a situation where collapse of the healthcare system is a strong concern due to growing cases of infection or an increase in public safety problems with the pandemic, consider evacuating overseas employees and their family members at an early stage.
  - Obtain information about charter flights to Japan, etc. from local Japanese consulates, Japanese Associations, and other relevant organizations as appropriate.
  - Prepare protocols in case employees must remain in the country or the evacuation is deemed too difficult to carry out, and develop a system that will enable the launch of a business continuity plan (BCP) based on domestic measures.

### 6.4 Important information you should know regarding international travel

#### 6.4.1 Advisories on overseas travel by the Government of Japan

1. Check information on travel advice and warnings related to infectious diseases on MOFA’s Overseas Safety HP.
  - Infection risk level 4: Evacuate.
  - Infection risk level 3: Avoid all travel.
  - Infection risk level 2: Avoid non‐essential travel.
  - Infection risk level 1: Exercise caution.
2. Restriction measures after returning to Japan
  - Note that restriction measures after returning to Japan are different for travellers who are returning from Level 3 countries/regions and those returning from Level 2 countries/regions: the former are subjected to entry restrictions and the latter are subjected to enhanced quarantine measures based on the Immigration Control Act.
3. Recent changes to infection risk level
  - MOFA has issued a Level 3 travel advice & warning for more than 100 countries. Meanwhile, the risk level has been lowered from Level 3 to Level 2 for countries where spread of the disease has started to slow down. (October 30, 2020)
  - As of October 30, the risk level has been lowered to Level 2 for the following nine countries: Thailand, Vietnam, Taiwan, Singapore, South Korea, China (including Hong Kong and Macau), Brunei, Australia, and New Zealand.

#### 6.4.2 Phased measures for resuming cross‐border travel

**FIGURE 1:**
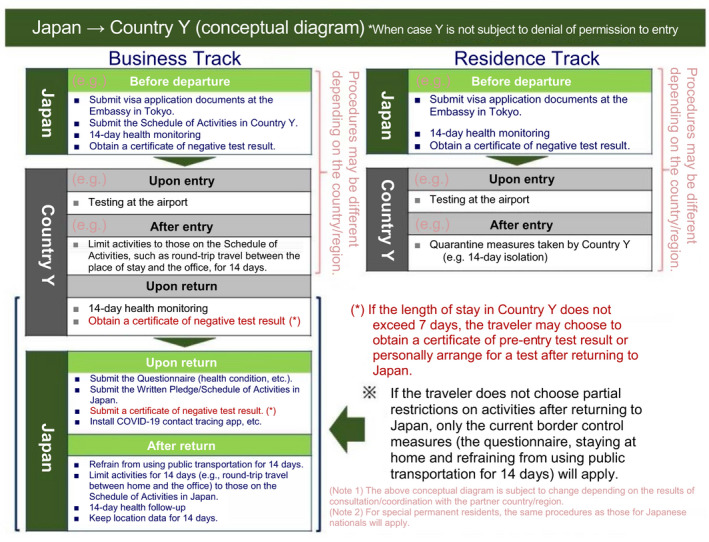
Conceptual Diagram of Business Track/Residence Track (for regions not subject to denial of permission to entry). For specific testing methods and designated medical institutions in the destination country, make sure to confirm with the Consulate of Japan in the region of stay or the Embassy of Japan in the destination country

**FIGURE 2:**
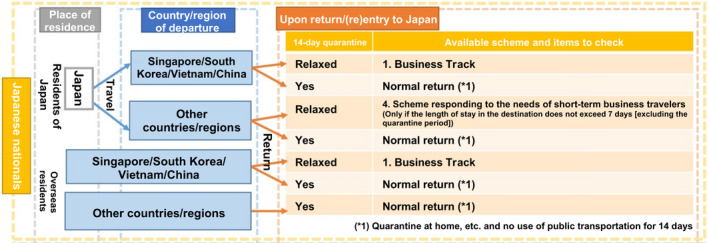
Conceptual diagram of schemes that can be used when returning to or re‐entering Japan

1. Travel to the countries/regions covered under Business Track/Residence Track (Figure 1)
  - This measure allows the entry/exit of personnel necessary for conducting business based on a bilateral agreement between Japan and its counterpart country. Table 14 shows the countries/regions with which the operation has started.
  - Business Track is a category that allows business activities limited to those described in the "Schedule of Activities" during the 14‐day self‐isolation period imposed immediately after entry into the destination country and upon return to Japan, and is intended for short‐term business travelers. It is also applicable when a Japanese living in a country covered under Business Track temporarily returns to Japan.
  - Residence Track is a category in which the 14‐day self‐isolation imposed after entry into the destination and upon return to Japan is maintained, and is intended mainly for long‐term residents, such as expatriates dispatched or placed on a rotation.
2. Travel to countries/regions NOT covered under Business Track/Residence Track
  - Check for restrictions on entry and post‐entry activities imposed by the destination country on the MOFA website.
  - Even when no restrictions on entry are described, it is possible that there are still entry requirements, such as having no symptoms, carrying a certificate of negative test result, being pre‐registered with the country's authorizing agency, etc. Therefore, confirm with the Consulate of Japan in the region of stay or the Embassy of Japan in the destination country.
3. Return/re‐entry to Japan (Figure 2)
  - When returning from a country covered under Business Track (including temporary return of a long‐term traveler), restrictions on activities are relaxed if the individual follows the specified procedures described above (Table 14).
  - Travelers who visited an area subjected to entry restrictions (Level 3) or reinforced border control (Level 2) within the past 14 days must comply with the following measures before and after returning to Japan:
    - Stay at the place designated by the Quarantine Station Chief (such as home) for 14 days after arrival and refrain from using public transportation, including transportation from the airport.
    - Secure the place of quarantine and method of transportation from the airport to the place of quarantine before arrival and report to the Quarantine Officer.
    - All travelers who returned from areas subjected to entry restrictions (Level 3) are required to undergo SARS‐CoV‐2 testing at the airport and a 14‐day health follow‐up by a public health center (use of LINE, etc. is also acceptable) upon return/re‐entry to Japan.
  - Japanese nationals residing in Japan and holders of residence status in Japan returning from a short business trip to any country/region (when the length of stay excluding the period of mandatory isolation does not exceed 7 days) are eligible for partial restrictions on activities upon return/re‐entry into Japan if certain conditions are met. In this case, the company accepting the person is responsible for conducting health follow‐up and other requirements for 14 days after arrival in Japan.
4. Certificate of pre‐entry SARS‐CoV‐2 test result
  - SARS‐CoV‐2 test required for the issuance of a certificate is performed before departure according to the method specified by the government of the destination country.
  - The current mainstream testing method is a PCR test, and is often required within 72 hours before departure.
  - Information on medical institutions that provide tests can be obtained from TeCOT (COVID‐19 Testing Center for Overseas Travelers) or the JSTH website (Table 15).
  - Note that some countries like South Korea, Singapore, and Vietnam designate medical institutions registered in TeCOT, while others such as China and the state of Hawaii designate medical institutions independent of TeCOT.
  - Make sure to obtain the latest information on the destination country's testing requirements and designated medical institutions from the Embassy/Consulates of Japan in the destination.

**TABLE 14:** Countries/regions with which the operation has started (as of November 30, 2020)

Business Track
*Singapore, South Korea, Vietnam, China (excluding Hong Kong and Macau)*
Residence Track
Thailand, Vietnam, Malaysia, Cambodia, Laos, Myanmar, Taiwan, Singapore, *South Korea*, Brunei, *China (excluding Hong Kong and Macau)*

**TABLE 15:** TeCOT (COVID‐19 Testing Center for Overseas Travelers)

TeCOT is a center operated by the Ministry of Health, Labour and Welfare (MHLW) and the Ministry of Economy, Trade and Industry (METI). The website allows overseas travelers to find and request reservations at desired medical institutions that can provide adequate SARS‐CoV‐2 testing required by the government of the destination country. By November 2020, approximately 600 medical institutions were registered. Reservations can also be requested through a travel agency, etc. The website provides details about SARS‐CoV‐2 test certificates, as well as a wide range of information intended for overseas travelers and medical institutions

## 7 IMPORTANT POINTS CONCERNING LEGAL OBLIGATIONS OF BUSINESS OPERATORS

### 7.1 Consideration of measures based on the amended Act on Special Measures

Following an amendment to the Act on Special Measures for Pandemic Influenza and New Infectious Diseases Preparedness and Response (Article 1‐2 has been added to the Supplementary Provisions; hereinafter referred to as “amended Act on Special Measures”), COVID‐19 is now classified as a pandemic influenza and new infectious disease. This chapter explains a number of measures related to this Act. In terms of legal requirements, the key points are in Article 4 of the amended Act on Special Measures (Responsibility of Business Operators and Citizens) (Table 16).

**TABLE 16:** Article 4 of the amended Act on Special Measures (Responsibility of Business Operators and Citizens)

Business operators and citizens must strive to prevent pandemic influenza and new infectious diseases and cooperate by carrying out countermeasures against these diseases Business operators must consider the potential influence of the spread of pandemic influenza and new infectious diseases and strive to take appropriate measures in their business operations *Registered business operators* specified in Article 28, paragraph 1, item 1) must endeavor to continually provide medical care and services contributing to the stability of the lives of the citizenry and the national economy even in the event of pandemic influenza and new infectious diseases

As stated above, point 1 specifies the duty of business operators to strive to prevent infectious diseases such as COVID‐19 and to cooperate by implementing infection prevention measures, and point 2 specifies their duty to take appropriate measures concerning their business operations by considering the potential impact of the spread of the disease. In other words, business operators are legally required by the amended Act on Special Measures to make efforts to close offices and adopt telework. Additionally, they are to examine and implement measures to balance their business continuity and safety considerations based on the amended Act on Special Measures.

The registered business operators described in point 3 refer to “business operators engaged in the provision of medical care or services contributing to the stability of the lives of the citizenry and national economy,” which includes healthcare professionals such as doctors, nurses and pharmacists; nursing care staff at nursing facilities; public health nurses; midwives; persons engaged in the sale of ethical pharmaceuticals; transportation workplaces; broadcast/newspaper workplaces; banking, water and sewer services; various retail workplaces such as drug stores; and cremation, grave management, and ceremonial occasion services (MHLW Notice No. 369, 2013, etc.). In accordance with the amended Act on Special Measures, registered business operators are required to continue their workplaces even during the current pandemic.

Furthermore, once a state of emergency is declared by the head of the Disaster Management Headquarters (the Prime Minister) (Article 32 of the amended Act on Special Measures), it will become possible, based on Article 45, for local governments to request the public to voluntarily refrain from going out (paragraph 1) and request or instruct (order) the public to restrict the use of schools and theaters, etc. and suspend or limit special events (paragraphs 2 and 3). Business operators should implement telework and consider/provide alternative services (changes in business structure) when unable to use facilities such as theaters.

Business operators need to recognize that business continuation will be significantly restricted by application of the amended Act on Special Measures. Even so, these business operators are still required to comply with general regulations for the safety of their employees, business partners, clients, etc. (safety obligations, Article 5 of the Labor Contracts Act and Article 415 of the Civil Code) and exercise reasonable care for business continuity (duty of care of directors and officers, Article 330 of the Companies Act and Article 644 of the Civil Code). Under the restrictions enforced by the amended Act on Special Measures, business operators who are quick to suspend their operations without giving careful consideration to the possibility of business continuation (through key operations, telework, web conferencing, outsourcing, alternative services [change of business structure], etc.) may receive a warning notice for violating “the duty of care.”

### 7.2 Specific measures taken by business operators based on the amended Act on Special Measures

Therefore, business operators need to carefully develop their action plans from the following viewpoints:

1. Review key operations

The need to avoid the risk of COVID‐19 transmission in the workplace is forcing employers to consider temporarily suspending their business (voluntary restrictions on business activities, store closure, reduction of business hours, telework, etc.). Employers are also required to review the aspects of their business operations that need to be suspended, reduced, or continued by assigning ranks ranging from “immediate suspension” to “continuation” so that they can be prepared for any changes in situation. It is also important to give serious consideration to the implementation of "alternative services" that will allow employers to practically continue with their key operations. For example, possible alternative services may be retail sales, food delivery, and catering for restaurant workplaces, mail‐order sales for retailers, and online classes and home tutoring for cram schools and preparatory schools.

2. Response to customers and what/how to provide information

Employers will receive inquiries from customers about both safety measures and business continuity. Check with the public relations department and website administrator about whether the company's public relations system, including its website, is sufficient to properly respond to these inquiries. At the same time, it is also important to consider providing advance notice of possible business suspension (postponement) and continuing operations.

3. Response and explanation to employees, business partners, etc.

In addition to considering the safety of employees and customers, it is also essential that employers strive to build cooperative relationships with employees engaged in key operations, labor unions, and vendors such as supply chains to better overcome challenges related to continuing the business. In doing so, employers should set up opportunities to explain the companies’ actions so that trusting relationships will be maintained.

4. Cooperation with an occupational health physician

To fulfill their duty to consider the safety of employees, employers should seek professional support, such as by asking for medical advice and opinions from an occupational health physician. In particular, complaints of telework‐related mental health problems should be handled in cooperation with an occupational health physician. It is necessary to promote information sharing with the company's occupational health physician and have the physician establish a cooperative framework for COVID‐19 response plan in addition to normal occupational health activities.

5. Confirmation of internal controls across the company

Through these efforts, employers should check internal communication (internal controls) regarding response measures for COVID‐19 across the company, from management to employees (including the occupational health physician), and make modifications as needed.

6. Setting up COVID‐19 countermeasures headquarters in the workplace (an example)

To make adequate decisions in response to shifting situations, it is desirable to set up COVID‐19 countermeasures headquarters in the workplace to allocate decision‐making authority. A framework should be established to enable those authorized to make decisions about business suspension, telework, short‐time work, alternative services, etc. to confirm information with personnel at the countermeasures headquarters (such as personnel in charge of human resources management, risk management, public relations, and legal affairs, and the occupational health physician) via web conferencing, etc. to facilitate their decision‐making process.

### 7.3 Examination of legal considerations of telework

#### 7.3.1 Legal considerations of telework

1 Introduction

As a protective measure against COVID‐19 disease, telework may be ordered (or approved) to prevent transmission while commuting and spread of the disease in the workplace. However, employees infected with COVID‐19 may be at risk of worsening symptoms as a result of telework. When teleworking, it is difficult for the employer to sufficiently check on the employee's health condition, which raises concerns from a security obligation viewpoint. So, even if the infected employee claims to be able to work despite contracting the disease, the employee should not be ordered (or allowed) to work from home.

2 Overview of problems associated with telework

First, it should be clear that labor‐related laws such as the Labor Standards Act and employment rules also apply to telework. Therefore, it is necessary to ensure that the teleworking method complies with labor laws and that there are no discrepancies with employment rules. Next, since employees are geographically isolated from the workplace and supervisors, maintaining sufficient personnel management and information control can be a problem. The MHLW has published guidelines for the appropriate introduction and implementation of off‐site work using information and communications technology (hereinafter referred to as "Teleworking Guidelines"), which should be taken into consideration when making telework arrangements.

3 Compliance with labor laws
  - Management of working hours

When teleworking, it is difficult to determine the exact working hours due to geographical distance from supervisors and an inconspicuous distinction between work and private life. However, it is still necessary to manage the hours of work for employees who telework. The Teleworking Guidelines also state that employers are required to properly track the hours of work employees conduct from home based on the relevant guidelines. For example, employees’ computer usage needs to be monitored and properly recorded as an objective measure. Even when working hours need to be self‐reported, an investigation should be conducted as needed to check whether the reported work hours are consistent with the actual hours worked. If they are not, corrections must be made.

Therefore, employers must be aware that a system needs to be put in place to manage the working hours of remote employees. Attention should also be paid to the handling of work interruptions and breaks when working from home. Such non‐work time needs to be handled properly with reference to the Teleworking Guidelines and other resources. Since an employees’ home is located outside the workplace, a problem may arise as to whether the System of Deemed Working Hours Outside the Workplace can be applied. In this system, employees are considered to have worked for a statutory number of working hours, regardless of the hours actually worked. However, this system is considered applicable only if "it's difficult to calculate the number of hours worked."

Many companies probably provide their employees with company mobile phones or computers to access work systems to facilitate efficient teleworking. In such cases, the System of Deemed Working Hours Outside the Workplace is most likely not applicable. Since employers are also required to manage their employees’ working hours from a security (health) obligation viewpoint to prevent overwork, it is necessary to monitor the hours worked by employees in management positions and those subject to the discretionary labor system or the System of Deemed Working Hours Outside the Workplace. Regarding the prevention of overwork, it may be more difficult not to overwork when working from home. For remote employees, prohibiting overtime and holiday work altogether may be worth considering.

- Information management

Without the direct involvement of supervisors and colleagues, the risk of information leakage is considered higher when working from home than working in the office. Therefore, when ordering (approving) telework for employees who work with confidential information, employers need to ensure that they have established information management policies, etc. to reduce the risk of information leakage.

#### 7.3.2 Need for adjustments in employment rules, etc.

When ordering or approving telework for a temporary reason such as the COVID‐19 pandemic, there is no need to revise company employment rules. Although in some companies, employment rules may specify the places of work, it is possible to order telework in accordance with rules pertaining to business trips or transfer orders.

However, telework incurs expenses normally not needed when working in the office (cost of equipment, telecommunication, utilities at home), but also eliminates the need for commuting. Handling of these necessary and unnecessary expenses needs to be considered. Therefore, it is advisable to establish telework policies, etc. as a supplement to employment rules to specify the details.

## DISCLOSURE

*Approval of the research protocol*: N/A. *Informed consent*: N/A. *Registry and the registration no. of the study/trial*: N/A. *Animal Studies*: N/A. *Author contributions*: all authors worked cooperatively to complete this guideline. *Conflict of Interest*: N/A.

## Supporting information

Supplementary Material
