# Supplementary material for: A Guide for Businesses and Employers Responding to Novel Coronavirus disease (COVID‐19): 4th edition
Source: J Occup Health. 2021 Oct 29;63(1):e12225. doi: 10.1002/1348-9585.12225 (PMC8250361; doi:10.1002/1348-9585.12225)
Supplement: Supplementary file 1 — Supplementary Material [file JOH2-63-e12225-s001.pdf]

Appendix 1. Benefits, Wages, Leave Allowances, and Others

Benefits

Injury and Sickness Allowance (benefits under the Health Insurance Act, MHLW)

The injury/sickness allowance program provides income security to an insured person with health insurance (employee) who is absent from work due to being treated for non-occupational illness or injury. If an insured person (employee) has become infected with SARS-COV-2 for a non-occupational reason, that person will be paid a daily amount equivalent to 1/30th of the average monthly standard remuneration during the last 12 months (1/30th of the average monthly wage [A]) multiplied by 2/3 (B) as injury/sickness allowance. The number of days paid (C) is the period starting three days after the person became unable to work due to medical treatment until the day the person returns to work (up to one year and six months) (Figure 3).

Figure 3: **From the Health Insurance Act**

Total amount of payment = **1/30 of the average monthly standard remuneration during the last 12 months (A)** × **2/3 (B)** × **Number of days paid (C)**

During the pandemic, if an employee who was self-isolating at home due to subjective symptoms, such as a fever, was unable to visit a medical institution because of a deteriorated physical condition but started to recover the next day without medical care, that person may still be eligible for injury/sickness allowances, which is the noteworthy point of this benefit program (Office Communication of Health Insurance Division, Health Insurance Bureau, MHLW, dated March 6, 2020). In such cases, the submission of a document prepared by the employer certifying that “the insured person (employee) did not engage in labor during the specified period due to medical treatment” is required. The document will be reviewed, and once approved, the employee may be eligible to receive injury/sickness allowances. Depending on the insurer, another benefit known as the “injury/sickness allowance additional sum” may also be available, so employees are encouraged to ask about it.

Workers' Compensation (industrial accident compensation insurance, MHLW)

If an employee has been identified as having contracted COVID-19 through an occupational route, the employee is eligible to receive workers’ compensation. To claim workers’ compensation, contact the designated Labor Standards Inspection Office in the area for consultation (Refer to “5. Workers’ Compensation in Q & A on COVID-19 [for workers] [as of November 13, 2020]” on the MHLW website). A decision on whether or not an employee is eligible for workers’ compensation needs to be made appropriately by considering the current state of the COVID-19 pandemic and the potential of the disease to spread through an asymptomatic population. For this reason, the following is specified: “At present, in cases where the infection is highly likely to have been work-related and is recognized as being attributable to work, the employee shall be eligible for workers’ compensation, even if the route of transmission has not been identified by an investigation” (Labor Standards Bureau Compensation Division Notification 0428-1, April 28, 2020).
Under this premise, further explanations are provided, as follows:

- As a general rule, if a physician, nurse, or nursing care provider involved in the provision of consultation, medical care, or nursing care for a patient becomes infected with COVID-19, they shall be eligible for workers’ compensation unless the transmission is clearly unrelated to work.

- For workers who are not healthcare professionals, if the source of infection was obviously in the workplace (i.e. the route of transmission has been identified), the worker shall be eligible for workers' compensation.
- For workers who are not healthcare professionals and the above does not apply (i.e. the route of transmission has not been identified by an investigation), if the worker has performed work duties in a relatively high-risk environment, the decision as to whether or not the infection is highly likely to be work-related and is recognized as being attributable to work shall be made properly on a case-by-case basis.
- For workers who have traveled on business to a country where many COVID-19 cases have been reported, if the trip is objectively considered to have carried a high risk of COVID-19 infection, the decision as to whether or not the infection was contracted on the business trip shall be made on a case-by-case basis.

Employment Adjustment Subsidy (subsidies based on the Employment Insurance Act, MHLW)

Employment adjustment subsidies are meant to subsidize part of the leave allowances paid by business operators who have been forced to reduce their business activities due to the COVID-19 pandemic but have strived to keep their employees by having them take a leave of absence, receive educational training, or go to another company for secondment. The disbursement of the subsidy and eligibility should be applied to the leave of absence or alternative activities from April 1, 2020, to the last day of February 2021, as described below (Employment Adjustment Subsidy Guidebook).
According to a press report, the MHLW has been making adjustments to extend the special measures pertaining to the Employment Adjustment Subsidy beyond January 2022.

- Increasing the subsidy rate for leave allowances (4/5 for small/medium enterprises and 2/3 for large enterprises)
  - Increasing the subsidy rate for employers avoiding dismissing employees (10/10 for small/medium enterprises and 3/4 for large enterprises)
- Increasing the amount added for the implementation of educational training programs (2,400 yen for small/medium enterprises and 1,800 yen for large enterprises)
- Extending eligibility to those who have been insured and under continuous employment for less than six months, such as new graduates
- Making coverage available separately from the subsidies paid up to 100 days per year
- Making coverage applicable to the leave taken by employees not insured by employment insurance

Subsidy for Elementary School Closure, etc. (subsidies based on the Employment Insurance Act, MHLW)

Subsidies for elementary school closure, etc. are paid to support business operators who have granted paid leave (excluding annual paid leave under the Labor Standards Act) to parents (regular and non-regular employees) who are forced to take leave due to the temporary closure of an elementary school, etc. as a consequence of the COVID-19 pandemic. This coverage applies to business operators who have granted paid leave (full wages) separately from annual paid leave under the Labor Standards Act to employees who need to take care of their children in the following cases:

- The child attends an elementary school, etc. that has been temporarily closed due to guideline recommendations.
- The child needs to stay home from elementary school, etc. for reasons such as having contracted COVID-19
- Amount paid: An amount equivalent to the wages paid to eligible employees who receive paid leave x 10/10
  - The upper limit is 15,000yen per day (for leave taken after April 1,2020)
- Applicable period: For paid leave taken between February 27 and September 30,2020

- Excluding days when the school is supposed to be closed according to its regular calendar, such as spring and summer breaks.
- Application period: through December 28,2020 (or through March 31,2021, for leave taken between October 1 and December 31,2020)

For self-employed persons, etc.

Financial support is provided for self-employed parents who are unable to perform a contracted project because they need to take care of their child due to the temporary closure of an elementary school, etc. as a consequence of the COVID-19 pandemic. In such cases, a fixed amount of 4,100 yen per day (from February 27 to March 31, 2020) and 7,500 yen per day (From April 1 to September 30, 2020) shall be paid for the days when the worker was unable to work.
Application period: through December 28, 2020 (or through March 31, 2021, for missed workdays between October 1 and December 31, 2020)

Housing Security Benefit (independence support system for people living in poverty, MHLW)

The Housing Security Benefit Support Project has been traditionally implemented to provide benefits to those who have lost their job in the last two years due to dismissal or bankruptcy. Considering the current situation as a consequence of the COVID-19 pandemic, eligibility for involvement in this project has been expanded to include those who have yet to lose their jobs but are at risk of losing their housing because of reduced incomes resulting from having to take leave or similar reasons. These individuals can receive an amount equivalent to their rent for a fixed period of time on the condition that they conduct a job search faithfully and diligently. With this extension of eligibility to those whose incomes have decreased due to having to take leave, the payment from July 2020 will be calculated using a different formula from the one previously used (if the household income exceeds the standard amount, "the standard amount + the amount of rent - the amount of the household income" will be paid [with the maximum amount of housing aid]).

- Payment amount (approximate amount in special wards of Tokyo): Single-person household, 53,700 yen; 2-person household, 64,000 yen; 3-person household, 69,800 yen
- Payment period: 3 months (extension allowed up to two times, for a maximum of 9 months)

Sustainable Benefits for Business Continuity (FY 2020 Supplementary Budget Administrative Project of Sustainable Benefits for Business Continuity by the Small and Medium Enterprise Agency, METI)

For business operators who are significantly affected by the voluntary suspension of sales activities, etc. due to the spread of COVID-19, sustainable benefits are provided to support their business continuation and recovery and can be used widely for all areas of business.

- Those eligible for the benefits: Small and medium-sized enterprises (≤2,000 full-time employees). Not applicable to large companies with a capital of ≥1 billion yen. Juridical persons other than companies, such as medical corporations, agricultural corporations, and NPOs, are also eligible.
- Payment amount: The amount should not exceed 2 million yen and is calculated as follows: total sales revenue in the previous year – sales in the applicable month multiplied by 12 (in units of 100,000 yen; fractions less than 100,000 yen should be rounded down).
- Requirement: A month in which the business faced a decrease in sales by ≥50% year-on-year due to the COVID-19 pandemic after January 2020 (subsequently “applicable month”)
- Application period: May 1, 2020, to January 15, 2021

Wages and leave allowances

Is it necessary to pay wages to employees who are self-isolating at home?

1. When employees voluntarily stay home from work due to having symptoms, such as a fever
In labor agreements, the provision of labor is the prerequisite to the right to claim wages by an employee ("no work, no pay" principle). Therefore, if an employee voluntarily missed work due to symptoms, such as a fever, without a diagnosis of COVID-19, the employee did not provide labor services and therefore may be considered to have no right to receive wages or leave allowances during the period based on the principle of "no work, no pay." The response to Q2 regarding "2. Leave allowances and annual paid leave under the Labor Standards Act in Q & A on COVID-19 (for workers) (as of November 13)" says that "a worker who takes voluntary leave of absence due to symptoms, such as a fever, without a diagnosis of COVID-19 is not eligible to receive leave allowances." Therefore, the general consensus appears to be that such workers are by nature not entitled to wage compensation (for these workers, there is the aforementioned injury/sickness benefit program).
However, despite the fact that central and local governments have been requesting employees with a fever or other symptoms to stay home in order to prevent the spread of the disease, no wages or leave allowances are paid to employees who actually comply with the request, as their actions are based on free will; this situation is hard to accept for such employees, and in such cases, employees may be tempted to hide their symptoms in order to come to work, which can result in the spread of the disease in the workplace, bringing about substantial damage to the company.
Therefore, for employees who take a voluntary leave of absence due to symptoms, such as a fever, some form of compensation should be considered, such as a certain allowance (e.g. setting up a special paid sick leave program), on the condition that they record their symptoms and regularly report to the company.

2. When the company orders employees to stay home (legal handling)
As mentioned above, in labor agreements, the provision of labor is the prerequisite to the right to claim wages by an employee ("no work, no pay" principle). By this logic, employees in self-isolation do not provide labor services and therefore should not be able to claim wages during the isolation period. However, there are some cases in which wages or leave allowances must be paid to such employees, as shown below.
<u>Article 536, paragraph 2 of the Civil Code</u>
If the performance of any obligation (note: providing labor) has become impossible for reasons attributable to the obligee (note: employer), the obligor (note: employee) shall not lose his/her right to receive performance in return (note: wages).

⇒ In cases involving any "reason attributable to the obligee", the employer must pay wages, even if no labor has been provided.

<u>Article 26, Labor Standards Act</u>
In the event a worker is absent from work for reasons attributable to the employer, the employer shall pay an allowance equal to at least 60% of the worker's average wage to each worker concerned while they are absent from work.

⇒ In cases involving any "reason attributable to the employer", the employer must pay at least 60% of the worker's average wage, even if no labor has been provided.

What must be clarified, then, is whether or not self-quarantine for reasons related to the COVID-19 pandemic is considered a "reason attributable to the obligee" in paragraph 2 of Article 536 of the Civil Code or a "reason attributable to the employer" in Article 26 of the Labor Standards Act. First, while the "reason attributable to the obligee" in paragraph 2 of Article 536 of the Civil Code and the "reason attributable to the employer" in Article 26 of the Labor Standards Act may be similar terminologically, they have different meanings. The "reason attributable to the employer" in Article 26 of the Labor Standards

Act has a broader meaning and is considered to include managerial and administrative issues on the employer's end (Northwestern Airlines Case, Supreme Court, July 17, 1987, Journal of Labor Cases No. 499, p. 6). Furthermore, in administrative interpretation, a subcontracting factory suspending its operations due to being unable to acquire materials and funds because of financial difficulties experienced by the parent factory is considered to be attributable to the employer of the parent factory (June 11,1948, Labor Standards Bureau Director Notice No. 1998).

Examination of specific cases

1. If an employee has become infected with COVID-19

An employee who has contracted COVID-19 may become incapacitated and is likely to become a source of workplace transmission (cluster infection). Therefore, the employee is considered unable to work. If the infection is not related to the employee's duty or workplace, it is not considered "attributable to the obligee" or "attributable to the employer." Therefore, the employer is not required to pay wages to the employee. However, if the COVID-19 is related to the employee's duty or workplace (arising out of employment/in the course of employment [AOE/COE], e.g. transmission in the workplace), and the employer has not implemented necessary measures to prevent infection, it is considered "attributable to the obligee," and the payment of full wages is deemed necessary.

2. If an employee who has symptoms, such as a fever, is ordered to stay home

When an employee has symptoms, such as fever, but is not incapacitated at work, the legal basis for placing such an employee on leave at the employer's discretion is that the employer has the obligation to consider the safety of the employee and protect other employees from the risk of virus transmission. In such cases, there is basically no "reason attributable to the obligee." However, the employer's managerial/administrative needs (need to fulfill the obligation of considering workplace safety) are recognized as "reasons attributable to the employer." Therefore, the payment of leave allowances is deemed necessary.

If an employee has been identified as suspected COVID-19 by the employee's "primary physician/nearby medical institution" or "COVID-19 consultation service counter provided by a local government," or if the employee was instructed by the company to visit the abovementioned facilities for consultation but did not comply, there is no "reason attributable to the obligee or the employer," and the payment of wages is thus considered unnecessary, as in above (1).

If the employee has been identified as having contracted COVID-19 due to a reason related to the employee's duty or workplace (AOE/COE) (e.g. transmission in the workplace) and the employer has not implemented necessary measures to prevent transmission, it is considered that there are "reasons attributable to the obligee," and the payment of full wages will be necessary as described before.

3. If an employee is ordered to stay home because a family member in the same household has contracted COVID-19

Even if someone in the same household has contracted COVID-19, if the employee has not been identified as having been infected with the disease but is ordered by the employer to self-isolate (as a close contact), the order is perceived as being born out of a need to consider the safety of other employees, which is not entirely considered "a reason attributable to the obligee," thereby not requiring the payment of full wages to the employee. Even so, the isolation order is recognized as managerial/administrative needs on the employer's end and is considered "attributable to the employer." Therefore, the payment of leave allowances is deemed necessary.

4. Employees who have returned from COVID-19-high-risk regions
For example, employees returning from regions at high risk for COVID-19 transmission, such as one for which a travel advisory warning has been issued by MOFA, may have been infected with the disease. If the reason for visiting the region was to perform work duties (such as a business trip), the case is considered “attributable to the obligee and the employer,” and the payment of full wages is required. However, if the reason for the visit was not related to performing duties (such as private trip), the case is considered the same as (3). Self-isolation in such instances is ordered out of consideration of the safety of other employees, which cannot be perceived as “a reason attributable to the obligee.” Therefore, the payment of full wages to the employee is not required. Even so, the self-isolation order is recognized as managerial/administrative needs on the employer’s end and is therefore considered “attributable to the employer.” For that reason, the payment of leave allowance is deemed necessary.

Points to note when an employer is forced to suspend business operations due to the spread of COVID-19

During the COVID-19 pandemic, a company may need to close its office or suspend operations as part of measures to prevent transmission of the disease. In such cases, employees who have not been infected with COVID-19 (i.e. employees who are able to work with no issue) are also forced to stay home, and whether or not wages should be paid to such employees becomes a problem.

1. When voluntarily suspending business, etc.
A company deciding voluntarily to suspend its operations, etc., does not mean that continuation of the business is no longer possible, unless it has become difficult to secure a sufficient number of employees for operations to continue. Under such circumstances, forcing employees who are able to work to stay home is considered “attributable to the obligee and the employer,” and the payment of full wages is deemed necessary.

2. When suspending business in response to a request made by the central or local government
Requests for business suspension by the central government, etc. are made to prevent the spread of COVID-19, which is expected to produce significant public benefits. Therefore, choosing not to comply with these requests can be quite difficult. However, such requests have no binding force. If an employer decides to comply with the request, the action is still considered voluntary, even though the employer is left with no choice but to suspend business operations. Therefore, in principle, the suspension is considered attributable to the obligee, as in (1) above, and the payment of full wages is deemed necessary.

3. When suspending business based on the Act on Special Measures for Pandemic Influenza and New Infectious Diseases Preparedness and Response
The Act on Special Measures for Pandemic Influenza and New Infectious Diseases Preparedness and Response (made applicable to COVID-19 by the latest amendment) states that prefectural governors can issue a request for the restriction/suspension of the use of theaters and other facilities visited by large numbers of people specified by a government ordinance (paragraph 2, Article 45 of the same Act), and if employers do not comply with the request without justifiable reason, the prefectural governors can order the restriction/suspension of the use of the aforementioned facilities "only when it is deemed particularly essential to prevent the spread of disease, protect the life and health of citizens, and avoid the spread of confusion in citizens’ lives and impairment of the national economy" (paragraph 3, Article 45 of the same Act).
In such cases, a request for use restriction/suspension based on the Act on Special Measures for Pandemic Influenza and New Infectious Diseases Preparedness and Response implies legal force. For

this reason, if the request for use restriction/suspension based on this Act has forced an employer to suspend their business, the reason for the office closure is considered “not attributable to the obligee or the employer,” thereby not requiring the employer to pay full wages to their employees. However, the employer is still required to pay wages for the work performed up to the office closure, and not doing so is obviously unacceptable.

4. When suspending business due to temporary closure of a partner company
Whether or not suspending one’s business due to the temporary closure of a partner company because of the COVID-19 pandemic is considered “attributable to the obligee or the employer” needs to be determined by performing a comprehensive assessment of the degree of dependency on the partner company, the possibility of utilizing alternative means, the length of time since the closure, and specific efforts made by the employer to avoid business suspension.
A court case ruled that the leave of absence resulting from terminated construction work by the prime contractor due to weather conditions was “not attributable to the employer,” and the payment of leave allowances (60% of the average wage) was not approved (Mogami Construction Case, Tokyo District Court, February 23). In that particular court case, the employment agreement stated that the allocation of work would be determined by the employer, and there was an understanding that the prime contractor could terminate the construction work depending on the weather, in which case no wages would be paid to employees. For these reasons, the court most likely decided that the loss of work was not “attributable to the employer” without questioning whether or not the employer had sought alternative means or made efforts to avoid putting employees on leave.

Maternal health care measures and wages for pregnant women (Equal Employment Opportunity Law, MHLW)

Pregnant employees may suffer great anxiety or stress over possible infection in the workplace, depending on the task assigned. Therefore, as part of maternal health care measures based on Article 13 of the Equal Employment Opportunity Law, a measure concerning COVID-19 disease has been additionally put in place (from May 7, 2020, to January 31, 2021). Thus, in cases where a worker’s psychological stress over COVID-19 associated with her work duties is deemed a risk to the health of the mother or fetus based on the health guidance or medical examination prescribed in the Maternal and Child Health Act and then reports this to her employer, the employer is required to make necessary accommodations for the worker based on the guidance from the worker’s physician or midwife, such as limiting her duties and on-site work (i.e. allowing her to perform telework or take a leave of absence).

However, the question remains as to how such women’s wages should be paid if shorter working hours/leave are allowed. In "’Q & A on Maternal Health Care’ in For Maternity Health Care for Female Workers (Equal Employment, Children and Families Bureau, MHLW, March 2007)," Q3 is: “How should we handle the wages for shorter working hours, breaks, and leave of absence?” To this, the response is as follows: "[Answer] It is advisable to determine such employees’ wages during shortened working hours, breaks, and leave by holding discussions between employees and management. According to a survey conducted by the MHLW in 2004, 46.7% of the companies that offer leave of absence for outpatient visits pay wages to the workers while they are on leave.” In "Considerations for pregnant workers, etc. to prevent the spread of COVID-19 in the workplace," a document issued to economic organizations by the Director of Health Service Bureau, etc. of MHLW as of April 1, 2020, the obligation of companies to pay leave allowances to workers stipulated in Article 26 of Labor Standards Act is mentioned. The document then says, “However, even when it is necessary to have workers take leave for unavoidable reasons related to COVID-19 and the payment of leave allowances is not required, please note that it is advisable to hold discussions between employees and management and incorporate a suitable policy, such as a

special paid leave program, into the employment rules so that the workers can receive leave allowances. In doing so, if an employer who has been forced to reduce business activities due to economic reasons instructs a pregnant worker to take leave, regardless of whether the employee is a regular or non-regular employee, the company may be eligible for the Employment Adjustment Subsidies. With that in mind, please thoroughly discuss the matter between employees and management to develop a policy that enables such workers to take leave free from worry." As clearly shown by these guidance and requests, employers may not be legally obligated to pay wages to pregnant workers on leave based on the principle of “no work, no pay”. However, employers should fully recognize the necessity of the measures that have been implemented this far and understand that ensuring that pregnant workers can take leave without worrying is most important. To that end, employers are expected to establish appropriate systems to pay wages and administer leave allowances to pregnant workers.

However, when a pregnant worker has requested a telework arrangement based on a physician’s written opinion and is instead ordered by the employer to take leave due to a lack of work that can be performed remotely, the principle of “no work, no pay” is not necessarily applicable. Paragraph 3 of Article 65 of the Labor Standards Act says, "In the event that a pregnant woman has so requested, an employer shall transfer her to other light activities.” Therefore, in principle, employers are required to assign light-duty work to pregnant workers upon request. During the COVID-19 pandemic, if a worker has requested a telework arrangement based on a physician’s written opinion, the worker needs to be placed on light duty at home. In such cases, the employer cannot just dismiss the request due to a lack of work that can be performed at home. If the employer is determined to not have made their best possible efforts to avoid a worker having to take leave, the situation may be considered “attributable to the obligee or the employer,” thereby requiring the payment of wages or leave allowances.

Use of special provisions when applying “guideline on debt workouts during a natural disaster” to the COVID-19 pandemic

A method of debt workout (establishment of specified conciliation for reduction and release of debts) using the "Guidelines on Debt Workouts for Victims of Natural Disasters," came into effect in April 2016. This method is intended to help individuals and self-employed individuals who have become subject to legal liquidation due to difficulty paying back debts, such as housing and business loans, as a result of loss of employment or a significant decrease in income/sales resulting from the COVID-19 pandemic, with the aim of assisting recipients in reconstructing their life and business. This method involves using special provisions when applying the “Guidelines on Debt Workouts for the Victims of Natural Disasters” to the COVID-19 pandemic (effective date: December 1, 2020). In these special provisions, the record date pertaining to the impact of COVID-19 is February 1, 2020 (date on which the government ordinance specifying COVID-19 as a designated infection [Government Ordinance No. 11, 2020] was enforced). These special provisions are applicable to the following debts:

1. Existing debts incurred before February 1, 2020
2. Debts caused by receiving the following types of loans to deal with decreased income and sales resulting from COVID-19 pandemic during the period between February 2, 2020 and the day of enactment of the special provisions (October 30, 2020):
    a. Special loan for the COVID-19 pandemic from a governmental financial institution
    b. Virtually no-interest/unsecured loans from a private financial institution
    c. Loans for individuals from a private financial institution

A debtor who is an individual meeting certain requirements may apply for debt relief (e.g. debt reduction, cancellation) under these special provisions. Specifically, the debtor will be assisted by registered support experts, such as a lawyer, tax accountant, real estate appraiser, etc., in negotiating with the creditor and eventually preparing special conciliation proposals for a summary court. These services rendered by

registered support experts are paid for by the government and are free-of-charge for the user. Since the use of this system will not be reported to a credit bureau and does not entail disadvantages, such as “not being able to qualify for a new loan,” it is advisable to consult with your creditor or contact the consultation service of a regional bar association.

Appendix 2. Issues with Conducting General Shareholders' Meetings

Measures and legal considerations for conducting general shareholders' meetings

Article 296 of the Companies Act stipulates that companies must convene shareholders by holding a general shareholders' meeting within a defined period of time after the end of business year. For this reason, a company has no choice but to hold a general shareholders' meeting, but how to ensure the safety of the shareholders, directors, and employees (COVID-19 prevention measures) requires planning. This section is dedicated to proposing measures that can be taken by a company for the above purposes. We ask each company to be prepared for changing situations and respond proactively by combining all or some of the measures proposed here or taking other measures.

1. Postponement of the general shareholders' meeting
The Companies Act does not necessarily require the calling of an annual general shareholders' meeting within three months after the end of each business year, and such meetings may be postposed to the following month. The Ministry of Justice has announced that, even when a provision in the articles of incorporation specifies when to call a general shareholders' meeting, if holding the meeting at the specified timing is not feasible due to the situation involving COVID-19, the meeting may be held within a reasonable period of time after the situation is resolved.

2. To postpone the general shareholders' meeting:
- If postponement is decided after sending out the meeting notice (i.e. if the date and time are different from those indicated in the initial notice), the convocation must be redone.
- If the meeting is postponed to the following month (beyond the record date), a new record date must be set, and a public notice must be made at least two weeks before the date.

However, for many companies, it is difficult to change the venue of the general shareholders' meeting, considering the work involved in finding another location, and some economic damage may be unavoidable. Furthermore, when postponing the meeting to the following month, notices of the record date must be sent out to ensure voting rights are appropriately exercised, in addition to notices of the record date for the distribution of dividends, if such matters are to be resolved at the meeting. Therefore, realistically, postponement of the general shareholders' meeting may be difficult for many companies.

3. Changing the venue of the general shareholders' meeting
As mentioned before, a request for suspension of the use of meeting/exhibition facilities has been issued as an emergency measure. As a result, the likelihood that hotels and other facilities that are typically used as venues for general shareholders' meetings will not be available to many companies is therefore high. Furthermore, under the ongoing state of emergency, holding a general shareholders' meeting where many shareholders and company executives and employees will gather together may be problematic from the perspective of safety, and such activities may be subjected to social criticism. Therefore, the meeting venue must be reconsidered (e.g. switching to the company's conference room). Regarding changing the venue, Q2 in "Q & A on Approaches to Conducting Shareholder Meetings" (hereinafter referred to as "shareholder meeting Q&A") dated April 2, 2020, and compiled by the Ministry of Economy, Trade and Industry (METI) and the Ministry of Justice (MOJ) indicated the following view: "If found unavoidable as a precautionary measure to prevent the spread of COVID-19, the size of the venue may be reduced or the number of shareholders who can attend may be limited to a reasonable extent by using the company's conference room or a similar site," and, "even if such efforts result in no shareholders attending, the general shareholders' meeting may still be conducted." In addition, Q3 in the shareholder meeting Q&A suggests the use of a pre-registration system to preferentially allow pre-registered attendees to enter the venue.

However, regarding the above Q2, the METI and MOJ expressed some reservations, adding “if found unavoidable” and “to a reasonable extent.” A court case concluded that shareholders being unable to enter the venue and thus unable to participate in the proceedings can be considered a valid reason for cancelling the general shareholders’ meeting altogether (Osaka District Court, March 28, 1974; Hanrei Times No. 306, p. 187). Therefore, careful consideration should be given to the reduction in the venue size and restriction of the number of shareholders who can enter the venue based on the pandemic situation and government announcements.

4. Adjourned shareholder meeting

On April 15, 2020, the Liaison Council for the Reponses to Account Settlement and Auditing Taking into Consideration the Impact of COVID-19 announced that, as an alternative to postponing the annual general shareholders’ meeting, holding two-stage meetings by hosting an adjourned meeting may be an option (e.g. holding the initial general meeting for the election of directors and similar business and holding the adjourned meeting for the presentation of financial statements, audit reports, etc.). Holding such two-stage meetings is suitable for a company that is able to hold a general shareholders’ meeting as scheduled but is unable to perform account settlement, auditing, etc., in a timely manner due to the impact of the COVID-19 pandemic. These two-stage meetings are also useful in that, even if the adjourned meeting is scheduled after the record date, a new record date need not be established.
However, no opinion has been expressed regarding the deadline by which the adjourned meeting should be conducted; therefore, careful consideration is needed concerning the implementation of two-stage meetings. In addition, if the distribution of dividends is to be resolved at the adjourned meeting scheduled after the record date, a new record date (and notice of the record date) will be needed, which should be noted with emphasis.

5. Guidance on promoting the exercise of voting rights in writing or via the Internet

Even if a company takes the abovementioned actions to change the schedule and venue or set up an adjourned meeting, protective measures against the spread of COVID-19 are essential when conducting a general shareholders’ meeting physically attended by shareholders. One potentially effective measure is to reduce the number of shareholders who attend the meeting. In particular, the active discouragement of attendance by high-risk individuals (such as the elderly and those with underlying disease) should be considered. However, the exercise of voting rights by shareholders should be regarded as important. To ensure the safety and protect the voting rights of shareholders, the company should promote the exercise of voting rights in writing or via the Internet on the company website and in the meeting notice. To encourage shareholders to vote by these methods, the following measures may be considered:

- Explain that the exercise of voting rights in writing/online is part of the measures to prevent the spread of COVID-19 and is socially meaningful.
- Explain that any so-called "souvenirs" conventionally provided to shareholders who attend the general meetings are not being planned this year.
- For shareholders who do not physically attend the general shareholders’ meeting, conduct a hybrid meeting (*details to be explained later).
- As a measure to actively promote the exercise of voting rights in wiring/online, offer a gift card or similar reward to shareholders who vote remotely using these methods.

and so on. As legal considerations pertaining to these measures may exist, consulting with a lawyer or similar professional is recommended before implementation.

6. Shortening the duration of the general shareholders’ meeting

In settings where many people engage in face-to-face interactions, such as carrying on conversations at a close distance for more than a brief period of time, the risk of spreading disease is high. Therefore, a

company should also consider shortening the duration of the general shareholders' meeting in order to prevent disease transmission. Shortening the speech time for shareholders (Q & A time) may be problematic in terms of ensuring directors' accountability (maintaining shareholders' right to ask questions) (Article 314 of the Companies Act). Therefore, reducing the time spent for report presentations (particularly video-based accounting explanations) is considered effective.
In Q5 in the shareholder meeting Q & A, the following view is indicated: "If found unavoidable as a precautionary measure to prevent the spread of COVID-19, taking reasonable measures while conducting the general shareholders' meeting" is possible. Specifically, as a measure to shorten the duration of time shareholders remain on site, the document suggests reducing the amount of time spent on proceedings and cancelling events such as social gatherings following the general meeting.

7. Measures to reduce infection risk

When conducting a general shareholders' meeting, caution should be exercised in securing the safety of the venue in order to protect not just shareholders but also the company executives and employees from the risk of contracting COVID-19. For example, the following precautions may be taken:

- Check the sanitation of the facility where the general meeting is held (e.g. doorknobs, seats, microphones, air conditioning/ventilation units, etc.). Furthermore, consider changing the seating arrangement (leave sufficient space between seats and place the seats for company executives and those for shareholders farther apart than usual). For the speaking microphone for shareholders, a stand microphone would be more appropriate than a hand microphone, which is meant to be handed from one person to another.
- As individuals in charge of the reception desk and site operation of the general shareholders' meeting are expected to speak face-to-face with many shareholders, take necessary precautions to prevent disease transmission, such as wearing a mask and similar personal protective equipment. Presenters should also wear a mask. There are cases in which the chairman may remove their mask during proceedings or company executives may remove their masks to deliver explanations to shareholders. Such mask removal may be done out of these individuals worrying about not adequately fulfilling their obligation to explain themselves to their shareholders; however, unless there are circumstances where it is particularly difficult to hear a voice through a microphone, masks should be worn at all times, even while speaking.
- At the entrance of the meeting venue, conduct temperature checks, make alcohol disinfectant solution available while encouraging attendees to use it, and ask them to wear a mask. In case of a shortage of masks or disinfectant solutions or similar sanitation supplies, the company needs to try to secure these supplies for the general shareholders' meeting themselves. This is also a reason why the remote exercise of voting rights should be promoted.
- Post a sign near the entrance of the meeting venue that reads: "Please let us know if you have a fever or feel unwell." This should also be communicated verbally by the people in charge. Even if no attendees report such symptoms, staff should proactively ask around and monitor attendees' conditions. Depending on the epidemic situation, consider conducting temperature check for attendees before entering the site. Q4 of the shareholders meeting Q & A indicates that it is also possible to "refuse the entry of shareholders with symptoms such as fever and cough or demand them to leave." However, not allowing these shareholders to exercise their voting rights entails risks. Therefore, it is preferable to ask for their participation in a separate room as much as possible by the methods described below:
- Ask shareholders in poor physical condition to participate from a separate room (a waiting room for shareholders should not be arranged this time, but a separate room should be prepared for said purpose with necessary accommodations, such as having a doctor in the room). However, the room

needs to be adequately equipped to give these shareholders the opportunity to speak. If a person shows symptoms reasonably suggestive of COVID-19, that person may be refused entry.

- During the Q&A session, shareholders will share a microphone; therefore, they should be asked not to remove their mask when speaking, and the microphone should be disinfected by wiping with an alcohol-based antiseptic solution after each use. When using a hand microphone, disinfect the grip as well.
- Plan measures to avoid congestion caused by people trying to exit the venue after the meeting. Consider measures such as dividing the seats into sections and allow people in only one section to leave at a time. Frequently ask for cooperation through the chairperson, facility information guide, etc.

8. Hybrid general shareholders' meeting

As a protective measure against COVID-19 relatively easy to implement, a hybrid general shareholders' meeting may be considered. From a legal standpoint, the hybrid general shareholders' meeting is a service wherein the meeting is broadcast/streamed for shareholders who do not attend the (actual) meeting. With the hybrid general shareholders' meeting, the same atmosphere experienced when attending the meeting in person can be conveyed, allowing remotely participating shareholders to hear opinions and responses to questions from board members, thus enhancing their satisfaction. Therefore, this measure is considered effective in reducing in-person participation to a certain degree. As communication failures are not considered a valid reason for cancelling the resolutions reached during the general shareholders' meeting, a hybrid meeting is considered a good preventive measure against COVID-19 that is relatively easy to adopt.

9. Fully online general shareholders' meetings

Virtual-only general shareholders' meetings are also expected to be realized. The current law (Companies Act) stipulates that the Board of Directors determines the "place" for a general shareholders' meeting and sends the notice of convocation. As an online platform is not considered a "place," virtual-only meetings are deemed impracticable. According to press reports, the government has started deliberation to allow companies to conduct virtual-only general shareholders' meetings. Although whether or not the government intends to revise the Companies Act, establish a special act, or examine the direction is unclear, virtual-only general shareholders' meetings are expected to become available by the end of 2021.

Appendix 3. Questions and Answers

COVID-19 general

(1) How long can the novel coronavirus survive outside of the body?

Reports have indicated that the virus can survive for several days on stainless steel and plastic, 3 hours in the air, and about 24 hours on cardboard. According to the MHLW website, the virus can survive up to 72 hours on plastic and up to 24 hours on cardboard.

(2) Can the novel coronavirus be transmitted from a person with no symptoms?

The virus is thought to spread through respiratory droplets, aerosol microdroplets, and close contact with an infected person. Some reports claim that the most contagious period is from two days before the onset of symptoms to immediately after the onset, and the virus can be spread by an asymptomatic person (an infected person with no symptoms). As such, COVID-19 may be contracted following exposure to an infected person who has yet to manifest symptoms. Furthermore, the virus is also known to exist in stool. Therefore, it is important to ensure you wash your hands after using the toilet.

(3) What is a cluster?

In the propagation of an infectious disease, a cluster refers to a small aggregation of infected individuals (infected population). Clusters have been confirmed in various part of Japan, such as in restaurants, live music clubs, exhibition facilities, welfare facilities, schools, and medical institutions. An infected person spreading the disease to another location links the clusters, causing an explosive increase in the number of infected cases. The MHLW has placed great importance on countermeasures against COVID-19 clusters.

Response to close contacts and infected persons

(4) An employee has been identified as a close contact by the public health center. Should I ask the employee to quarantine at home?

Once determined as a close contact, the person is instructed to undergo 14-day health observation and PCR testing by the public health center. Even if the test result is negative, health observation is still required. During the health observation period, the person is required to conduct hand-hygiene practice and wear a mask, pay attention to their physical condition, avoid non-essential outings, and, if traveling is unavoidable, refrain from using public transportation. Self-quarantine is preferable. However, when telework is not practical due to the nature of work and the employee needs to visit the office to perform duties, please make sure that the employee understands the importance of thoroughly following the abovementioned precautions and limiting contact with other employees before being allowed to enter the office.

(5) A family member of an employee has been identified as a close contact. The employee is asymptomatic. Can I allow the employee to come to work?

If a family member or someone residing in the same household was identified only as a close contact, the employee does not need to self-isolate. However, the employee is required to take strict precautions to prevent household transmission, such as wearing masks and practicing good hand hygiene. The employee also needs to pay attention to their own physical condition and, if they feel sick, refrain from coming to the office. In addition, depending on the PCR test result of the family member, the employee may be identified as a close contact. Therefore, the employee may be placed in self-quarantine until the family member’s negative PCR test result is confirmed.

(6) How do I respond to an employee who feels unwell but no fever?

Even without a fever, an employee who feels unwell may still have COVID-19. Therefore, please instruct the employee to stay home and not to come to the office. As a rough guide for when employees who feel unwell can return to work, please refer to "General return to work guidelines for employees who have not been tested for COVID-19” on p. 18 of this Guide. If they wish to return to work early, the employee should undergo SARS-CoV-2 testing at a medical institution.

(7) A family member of an employee has become infected with COVID-19 and has been self-isolating at home. As a close contact, the employee is currently self-isolating at home as well, but how long does the self-isolation need to last?

If a family member has become infected and been self-isolating at home, people residing in the same household are basically considered close contacts. The period of health observation of people residing in the same household as an infected person may be different depending on the designated public health center in the area (for more information, refer to Self-isolation on p. 4 of the document in the link), and they may be required to go through an additional 14-day health observation starting on the day when the infected person has been released from self-isolation.

(8) An employee has reported a fever that developed but subsided within 1 day. In such cases, is self-isolation at home "for 8 days after the onset of symptoms and until 72 hours have passed since the fever resolved and all other symptoms improved (without oral medication)” still necessary?

Some patients with COVID-19 may show only mild to almost no symptoms (not even a fever). We are also beginning to learn that a high proportion of cases are asymptomatic (infected persons with no symptoms). Even if a fever abates after 1 day, ruling out COVID-19 is difficult. Please advise the individual to self-isolate at home for the period of time specified in "General return to work guidelines for employees who have not been tested for COVID-19” on p. 18 in this Guide. If staying home (taking a leave of absence) for the above period is difficult in the employee’s circumstances, please advise them to receive SARS-CoV-2 testing as mentioned in the above (6). If such advice is not feasible, the employee may be allowed to return to work under the supervision of the employer provided “at least 72 hours have passed since the fever and other cold-like symptoms resolved without oral medications” as described in this Guide.

(9) How do I set criteria to allow an employee with COVID-19 to return to work?

For symptomatic cases, the patient can be discharged from the hospital if 1) 10 days have passed since the date of onset, and 72 hours have passed since the resolution of symptoms; or 2) the results of two PCR tests performed after the resolution of symptoms and at least 24 hours apart are negative. For asymptomatic carriers, the person can be discharged from the hospital if 1) 10 days have passed since the collection of a positive sample, or 2) 6 days have passed since the collection of a positive sample and the results of two PCR tests performed at least 24 hours apart are negative. When self-isolating at home or in a hotel, the criteria for hospital discharge need to be met to lift the self-isolation. Please determine the appropriate time for an infected employee to return to the workplace based on these criteria and by referring to the “general return to work guidelines for infected employees” on p. 19 in this Guide.

Environmental cleaning and disinfection

(10) How should environmental cleaning and disinfection be performed specifically?

To perform disinfection to reduce the risk of COVID-19, alcohol-based solutions (60%-95%) and sodium hypochlorite solution (0.05%) are effective. When performing disinfection, please use appropriate protective equipment, such as a face mask, gloves, gown, etc. After removing dirt from surfaces with a neutral detergent, wipe down commonly touched surfaces (e.g. doorknobs, handrails,

switches, faucets, etc.) in one direction with a paper towel soaked in disinfectant solution. Sodium hypochlorite is corrosive to metals. If used on metal surfaces, carefully wipe it off with a damp cloth. To disinfect toilets, use sodium hypochlorite (0.1%).

(11) If an employee has been infected in the workplace, should we perform cleaning and disinfection?

If COVID-19 case has occurred at work, ventilate the work area of the infected person and disinfect (by wiping down) the surfaces and objects the infected person may have touched with alcohol (60%-95%) or sodium hypochlorite (0.05%) disinfectant. Use sodium hypochlorite (0.1%) to disinfect toilets used by the infected person and areas where the person's bodily fluids, such as vomit, have been spilled. For this type of disinfection as well, use appropriate personal protective equipment (facemasks, gloves, gowns, etc.). For general guidelines concerning disinfection, please refer to "Workplace disinfection" in this Guide.

Use of face masks

(12) Should people without symptoms wear masks?

COVID-19 is most contagious from two days before the onset of symptoms until immediately after their onset. This means that an infected person can spread the virus to other people even before symptoms appear. Since wearing a face mask can prevent the release of virus-containing droplets while speaking, "mask wearing by everyone, including those without symptoms" is recommended. This concept is called universal masking and has been indicated in the Example of Practicing "New Lifestyle" by the Japanese government, with the WHO also recommending wearing masks on public transportation and similar public settings. However, please exercise caution when using a mask in the heat or during a high-intensity physical activity to avoid exhaustion resulting from breathing difficulty or discomfort caused by wearing a mask.

(13) Are medical masks (surgical masks) and general non-woven masks (for pollen) the same?

Both medical and general non-woven masks are made with non-woven materials. However, medical masks are designed to withstand use in medical settings and meet the criteria for Class I or Class II of the Medical Device Classification by the U.S. Food and Drug Administration (FDA) (such as bacterial filtration efficiency and fluid barrier protection). The testing methods for evaluating the performance of medical masks are specified by the American Society for Testing and Materials (ASTM). However, Japan has no criteria for assessing the performance of medical masks.

(14) Is it possible to reuse a mask?

Aside from those indicated as reusable, reusing a non-woven mask is generally not recommended, as the mask's performance is compromised by washing. Regarding the reuse of N95 masks, please refer to the information provided by the CDC or MHLW.

Roles of occupational health personnel

(15) Should face-to-face consultations provided by occupational health staff for employees be discontinued/postponed?

Consider the urgency and necessity of the consultation to decide whether or not to conduct it. Consultations should preferably be provided via Web conferencing. If Web conferencing is not available and a consultation needs to be conducted face-to-face, please take precautions to prevent disease transmission, such as by thoroughly practicing good hand hygiene, securing a sufficient distance between both parties while wearing masks, installing acrylic boards, ensuring adequate ventilation in the room, etc. If one of the parties shows a fever, respiratory symptoms, or other symptoms, please avoid conducting face-to-face consultations. For guidance on remote consultations by physicians, please refer to the Notice dated November 19, 2020.

(16) We have scheduled health checkup in our workplace. Should it be cancelled or postponed?

Health checkups required by the Industrial Safety and Health Act and other regulations should be conducted at medical institutions that have taken adequate measures to prevent transmission. When conducting health checkups, the "Three Cs" should be avoided in the facility by 1) keeping people with a fever and other symptoms from participating in health checkups, 2) practicing thorough hand hygiene and mask wearing, 3) limiting the number of people receiving health screenings at one time, and 4) ensuring adequate room ventilation.

Business trips

(17) How should I consider the evacuation criteria for expatriates?

In countries/regions with growing numbers of COVID-19 cases, movements of people and goods are restricted, and overseas employees and their families are required to act in accordance with the instructions of the local governments. With the rapid spread of the disease (characterized by increases in the number of new cases and deaths), we should expect possible restrictions on movement of people and the deterioration of medical situations in the affected country/region. For overseas employees who are high-risk individuals and their family members, please consider encouraging early evacuation. Particularly in regions that usually with low medical standards even in normal times and those categorized as Infection Risk Level 3, the medical situations may quickly deteriorate. As receiving the same level of medical care as in Japan may be difficult in such regions, please consider evacuating your employees by referring to the Travel Advice & Warning issued by the MOFA and information provided by Tabi Regi.

(18) COVID-19 cases are increasing globally. Do you recommend cancelling overseas business trips?

Please consider travel advisories issued by the MOFA and U.S. CDC, border control measures and movement restrictions after entry enforced on travelers from Japan, flight information, health condition of the traveler, medical situations in the place of stay, and other relevant factors when deciding whether or not to cancel a trip by weighing the need for the trip and the potential risks. If the traveler is considered to be a high-risk individual (linked to page 12) and the destination is located in a region categorized as Infection Risk Level 3, consider cancelling or postponing the trip. In addition, please make sure to advise the traveler to register with Tabi Regi.

(19) Should we be careful about anything in particular when supporting employees returning from high-risk countries (regions)?

When an employee returns to Japan from a country/region not covered under Business Track, please note that the restrictions after return differ depending on which country/region the employee stayed in, based on new measures for border enforcement: Level 3 countries/regions are subject to entry restrictions, while Level 2 countries/regions are subject to reinforced quarantine. However, when an employee returns to/re-enters Japan from a country/region covered under Business Track, restrictions on activities after return/re-entry can be relaxed by following certain procedures. Please consider whether or not travel to/return from the applicable country/region is necessary based on these situations.

(20) Do you recommend cancelling domestic business trips?

Refraining from traveling to or from a prefecture where the number of COVID-19 cases is rapidly increasing is recommended. To avoid the risk of infection, please preferentially use Web conferencing and similar tools.

(21) What are the precautions to take when traveling by airplane or bullet train?

In an aircraft, the air inside the cabin is replaced with fresh air every two to three minutes. Airplanes are also equipped with HEPA filters. In a bullet train, the ventilation system replaces the air inside with fresh air at least once every six to eight minutes. Therefore, the risk of infection is considered lower inside aircraft and bullet trains than in the general environment. However, as transmission through droplets and contact cannot be avoided altogether in such situations, all air travel passengers are currently required to wear masks by airline companies. Basic preventive measures, such as wearing masks and refraining from speaking loudly, apply to not just airplanes and bullet trains but all vehicles. Persons who develop any suspicious symptoms, such as coughing, are asked to refrain from their use. In the waiting room at the airport or train station, avoiding crowded spaces, wearing a mask, refraining from talking loudly, and maintaining a distance of 2 m from others whenever possible is recommended.

Responding to COVID-19 cases in the workplace (Onsite clinic)

(22) I examined a patient with suspected COVID-19 symptoms at the company clinic. What response measures should I take now?

Please instruct the patient to visit a medical institution where SARS-CoV-2 testing is available. Disinfect the area around the employee's desk and surfaces/objects that the employee may have touched using an alcohol-based disinfectant. The standard area to be disinfected is about a 2-m radius around where the person works. In case of a confirmed infection, please initiate contact tracing. Consider placing people with whom the employee has come in contact (contacts) in self-isolation even before any infection has been confirmed.

(23) After the above 22, the employee was confirmed as a positive COVID-19 case. No instruction has been provided from the public health center. What response measures should I take?

Please contact the public health center assigned to your area and wait for instructions on what actions to take. The health center will instruct you on the submission of certain information required to identify close contacts, so prepare that information while waiting for instructions. If any of the contacts identified in (22) are determined to be close contacts by the health center, notify those people that they are required to undergo PCR testing and 14-day health observation.

Service operations and meetings

(24) How should I change the policy on cafeteria use in my company?

Consider restricting the number of employees allowed in the cafeteria at once by changing the hours of use for each floor or taking similar measures to prevent overcrowding. Please encourage employees to take precautions, such as avoiding sitting right next to others, speaking while eating, eating in a hurry (as this can cause choking/coughing), and sitting directly across from others. Please make sure that these changes in the policy will not significantly shorten the lunch and break times for employees. At the time of preparing these guidelines, there are no reported cases of COVID-19 transmission via foods or food packages. However, foods served in a buffet style and sharing of tabletop seasonings may spread the disease. Please ensure thorough hygiene control and consider changing the method of food provision.

(25) What precautions can be taken to prevent virus transmission in customer service operations?

1. Measures for employees: Practicing good hand hygiene by handwashing is essential, and avoiding touching the face and eyes is important. Please prioritize safety measures for employees by having them wear face masks and make a conscious effort to maintain an adequate physical distance (2 m) from customers. If necessary, use face shields to prevent virus transmission through the eyes.

2. Measures for customers: Asking customers to cooperate in the prevention of COVID-19 transmission is also important. Possible measures to be taken include asking those feeling unwell not to enter, requiring the use of face masks, placing alcohol disinfectant solution at the entrance, and implementing physical distancing (2 m).

(26) Should we cancel meetings with external clients?

Meetings with external clients can lead to job requests placing a further burden on the company's infection prevention efforts. Please use Web conferencing whenever feasible. If a face-to-face meeting is unavoidable, please take precautions, such as advising strict adherence to hand hygiene, mask wearing by all attendees, physical distancing, restricting the number of attendees, and adequately ventilating the room.

(27) Are there any points to note when conducting an in-house meeting in the workplace?

Please decide whether to hold a meeting or not by considering its urgency and necessity. Preferably, approaches such as Web conferencing should be employed in order to reduce the opportunities for direct contact. If a face-to-face meeting is unavoidable, please take precautions, such as advising strict adherence to hand hygiene, mask wearing by all attendees, physical distancing, restricting the number of attendees, and adequately ventilating the room.

Amended Act on Special Measures (The Act on Special Measures for Pandemic Influenza and Novel Infectious Diseases Preparedness and Response)

(28) What is the amended Act on Special Measures?

The official name of the law is the "Act on Special Measures for Pandemic Influenza and New Infectious Diseases Preparedness and Response." COVID-19 is a designated infectious disease based on the Infectious Diseases Control Law, but it does not fall within the definition of "new infectious diseases." As such, new paragraphs have been added to the Supplemental Provisions of the Act to include COVID-19 in pandemic influenza and new infectious diseases" so that the law can be applied for two years (hereinafter referred to as the "amended Act on Special Measures"). The amended Act on Special Measures allows the Prime Minister to declare "a state of emergency" when the spread of the disease may have a serious impact on the Japanese peoples' lives and national economy. However, since there is also a possibility that the freedom of the people may be unjustly restricted, both Houses have adopted supplementary resolutions, such as requesting preliminary reports to the Diet.

(29) What measures will be implemented when a state of emergency is declared based on the amended Act on Special Measures?

The declaration of a state of emergency allows prefectural governors to take measures 1) to 3) below and issue ordinances concerning the sales or storage of foods and pharmaceutical products. Those who do not follow the ordinances may be penalized.

1. Requests for voluntary restraint of non-essential outings and restriction on the use of facilities such as schools (Article 45 of the amended Act on Special Measures)
2. Establishment of temporary medical facilities in case of shortages of hospitals, medical institutions, etc. (Article 48 of the modified Act on Special Measures)
3. Preservation of the rights and interests of patients with pandemic influenza and new infectious diseases (Article 57 of the amended Act on Special Measures)

(30) Will a company being required to continue its operations during a state of emergency affect the company's occupational health professionals?

Business operators required to continue their workplaces during a state of emergency are not allowed to suspend their services/operations, even amid the spread of the disease. For example, hospitals

and other medical institutions as well as designated public institutions and designated regional public institutions that are marketing authorization holders, manufacturers of pharmaceutical products, or distributors of pharmaceutical products are required to take necessary measures to ensure continued manufacturing and distribution of medical and pharmaceutical products, medical devices, and regenerative medicine products in accordance with their business operational plans. These business operators need to maintain and secure the personnel needed for their business continuation. As such, these workplaces need to prepare in advance by implementing infection prevention measures, developing protocols to follow when a staff member is positive for COVID-19, establishing systems to allow for continued services/operations, securing necessary personnel, etc. Occupational health professionals are expected to actively provide support, training, and advice for infection prevention measures.
